# Bovine hepatic miRNAome profiling and differential miRNA expression analyses between beef steers with divergent feed efficiency phenotypes

**DOI:** 10.1038/s41598-020-73885-5

**Published:** 2020-11-09

**Authors:** Robert Mukiibi, Dayle Johnston, Michael Vinsky, Carolyn Fitzsimmons, Paul Stothard, Sinéad M. Waters, Changxi Li

**Affiliations:** 1 Department of Agricultural, Food and Nutritional Science, University of Alberta, Edmonton, AB T6G 2P5 Canada; 2 Animal and Bioscience Research Department, Teagasc, Grange, Dunsany, County Meath Ireland; 3 Lacombe Research and Development Centre, Lacombe, Agriculture and Agri-Food Canada, Alberta, T4L 1W1 Canada

**Keywords:** Gene expression profiling, Gene expression

## Abstract

MicroRNAs (miRNAs) are small RNA molecules involved in regulation of multiple biological processes through modulating expression of their target genes. Here we employed RNAseq to profile liver tissue miRNAome of 60 steers from Angus, Charolais, and Kinsella Composite (KC) populations. Of these animals, 36 animals (n = 12 for each breed) were utilized to identify differentially expressed (DE) miRNAs between animals with high (n = 6) or low (n = 6) phenotypic values of residual feed intake (RFI), a common measurement of feed efficiency. At a threshold of fold-change > 1.5 and P-value < 0.05, we detected 12 (7 up- and 5 downregulated in low-RFI animals), 18 (12 up- and 6 downregulated), and 13 (8 up- and 5 downregulated) DE miRNAs for Angus, Charolais, and KC steers, respectively. Most of the DE miRNAs were breed specific, with *bta-miR-449a* and *bta-miR-AB-2* being differentially expressed in all three breeds. The predicted target genes of the identified DE miRNA are mainly involved in cell cycle, cell death and survival, cell signaling, cellular growth and proliferation, protein trafficking, cell morphology, cell-to-cell signaling and interaction, cellular development, molecular transport, post-translational modification, as well as nutrient metabolism (lipids, carbohydrates, protein and amino acid). Our results provide insights into the bovine hepatic miRNAome and their potential roles in molecular regulation of RFI in beef cattle.

## Introduction

Genetic selection and breeding for more feed efficient beef animals is of great interest to beef producers, since increased feed efficiency will reduce the cost of beef production as feed and feeding related expenditures contribute up to 75% of the total variable production costs^1^. Additionally, studies have shown that breeding for more feed efficient animals can potentially reduce methane emissions from beef cattle^2,3^, which could consequently lower the environmental carbon footprint of beef animals. Understanding the genetic control of complex traits such as feed efficiency can help enhance the rate of genetic improvement of the traits via the designing of more effective genetic or genomic selection tools^4^. In this regard, multiple genome wide DNA marker association studies^5–9^, and global transcriptomic profiling studies^10–18^ have endeavored to identify markers, genes and biological functions associated with feed efficiency in beef cattle.

MicroRNAs are a group of small RNAs with an average length of approximately 22 nucleotides, mainly biosynthesized through the enzymatic cleavage of longer RNA molecules by DROSHA (nucleic) and DICER (cytoplasmic) RNase endonucleases^19–21^. The synthesized miRNA then combines with Argonaute proteins to form a miRNA-induced silencing complex (miRISC)^22^, which under the guidance of the miRNA predominantly binds to the seed regions in the 3′ untranslated regions (UTRs) of the target mRNA, and hence leading to down-regulation of the target gene^22,23^. In mammalian cells, miRNAs target and regulate expression of up to 60% of the transcribed genes^24^. Therefore, they are involved in multiple biological functions including cell proliferation, cell cycle, cell development, apoptosis, metabolism of amino acids, metabolism of lipids, metabolism of carbohydrates, and metabolism of minerals and vitamins^25^. In the liver, miRNAs have been implicated in regulating hepatic cell proliferation, hepatic metabolism of nutrients (including lipids, carbohydrates, vitamins and minerals, and proteins and amino acids), energy metabolism and detoxification^26^.

Previous transcriptomic studies have revealed the involvement of hepatic tissue in the molecular control of feed efficiency through identification of differentially expressed genes between efficient and inefficient animals^10–12,14,15,17,18^. For instance, in our most recent studies we detected multiple differentially expressed genes associated with feed efficiency traits, some of which are involved in key hepatic functions such as lipid metabolism, energy production, amino acid metabolism, and carbohydrate metabolism in beef cattle^18,27^. However, studies that have sought to identify possible miRNA regulation of genes for feed efficiency in beef cattle are limited^28–30^. Therefore, in this study we aimed to profile the hepatic miRNAome of beef steers from three beef breed populations including Angus, Charolais, and Kinsella Composite (KC), and to identify miRNAs associated with residual feed intake (RFI), a common measurement of feed efficiency, through small RNAseq differential expression analyses.

## Results

### miRNA sequence data and alignment quality

**Figure 1:**
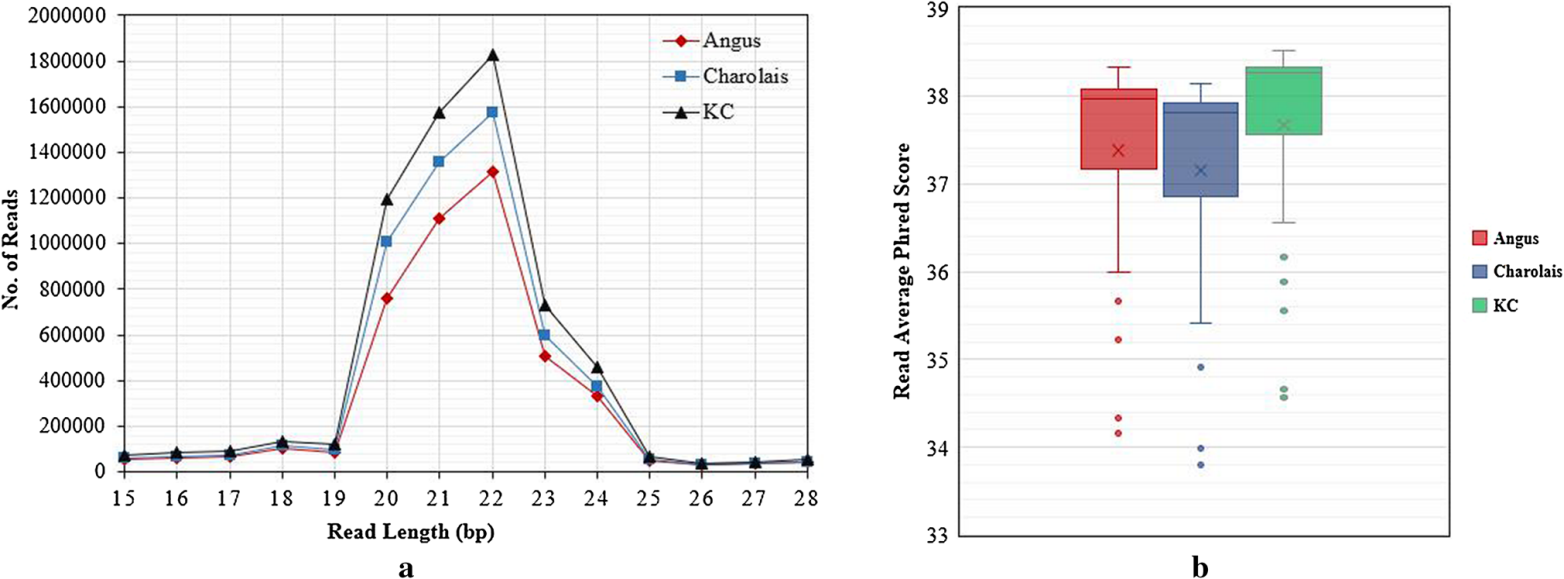
(**a**) Line plot showing the read length distributions in the final cleaned sequence data after quality control involving 3′ Illumina sequencing adaptor clipping, removing very long reads (> 28 bp) and short reads (< 15 bp), and removing reads that mapped to other small RNA species (rRNAs, snRNAs, tRNAs and snoRNAs) for Angus, Charolais, and Kinsella Composite (KC) samples; (**b**) Box plots showing the average Phred quality scores of the retained reads.

On average the Illumina next generation sequencing yielded over 9 M (million) high quality raw reads per sample for Angus and Charolais, and over 11 M for KC samples (Table S1 in Supplementary File 1). After 3′ adaptor clipping, an average of 36.78% of the reads were removed as long reads (> 28 bp), and an average of 8.19% of the reads were removed as short reads (< 15 bp) (Table S1 in Supplementary File 1). Additionally, on average 0.24%, 0.14%, 0.03%, and 0.03% of the reads were removed since they aligned to bovine rRNAs, tRNAs, snRNAs, and snoRNAs, respectively (Table S1 in Supplementary File 1). An average of 5.5 M reads were retained for miRNA profiling analysis by mirDeep2 (Table S1 in Supplementary File 1). Most of the reads ranged between 20 and 24 bp in length as shown in Fig. 1a, with an average length of 21 bp. The retained reads were of high quality as depicted by high average Phred scores in Fig. 1b. Of the retained reads, 74.78% (SD = 2.31%) mapped to the UMD3.1 bovine reference genome on average, ranging from 72.47% (SD = 1.2%) for Charolais to 77.09% (SD = 1.34%) for KC (Table S1 in Supplementary File 1).

### Known miRNA expression and novel miRNA profiles

**Figure 2:**
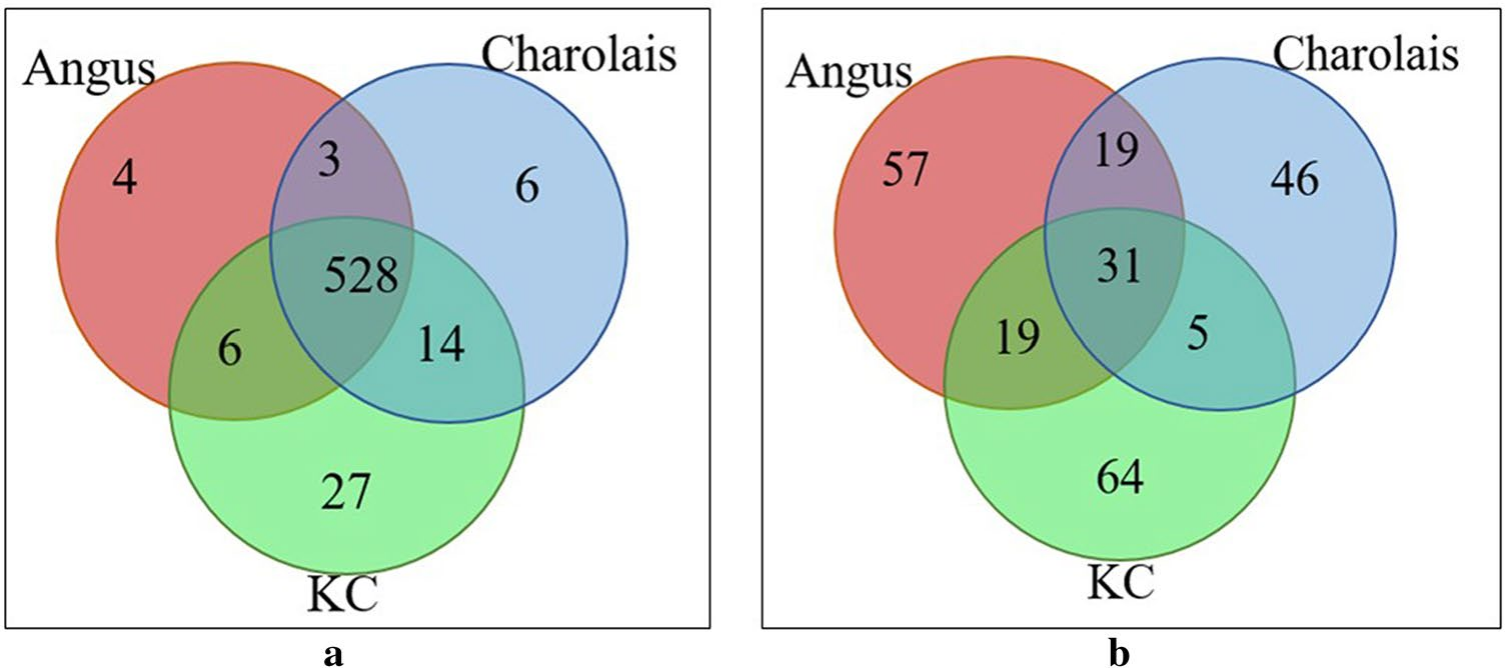
(**a**) Venn diagram showing overlap of expressed known miRNAs in the liver tissue of steers from the three studied breeds [Angus, Charolais, and Kinsella Composite (KC)]; (**b**) Venn diagram showing overlap of novel miRNAs identified between the three studied breeds (Angus, Charolais, and KC).

We identified 541, 551, and 575 expressed known miRNAs in Angus, Charolais, and KC samples, respectively. Of all these, 528 unique known miRNAs (approximately 90%) were common to all the three breeds as shown in Fig. 2a. Among the highly expressed miRNAs, *bta-miR-192* was the most abundant miRNA in all the three breeds with an average of 867,342, 1,060,828, and 1,272,798 aligned reads per sample from Angus, Charolais, and KC populations, respectively. Ten miRNAs showed predominantly high expression including *bta-miR-192, bta-miR-143, bta-miR-148a, bta-miR-26a, bta-miR-30a-5p, bta-miR-22-3p, bta-miR-27b, bta-let-7f, bta-miR-27a-3p,* and *bta-miR-101,* and they accounted for an average of 78.4%, 78.3%, and 77.9% of the total aligned sequence reads in Angus, Charolais, and KC animals, respectively. The top 20 expressed miRNAs across studied samples from each of the breeds are presented in Table 1, while all the expressed known miRNAs identified, and their average aligned read counts in each breed are presented in Supplementary File 2.

**Table 1:** Top 20 highly expressed known miRNAs (by aligned read counts) from each of the three breed populations (Angus, Charolais, and KC) studied.

	Angus		Charolais		Kinsella composite (KC)	
	Expressed known miRNAs	Average count/sample	Expressed known miRNAs	Average count/sample	Expressed known miRNAs	Average count/sample
1	***bta-miR-192***	867,342	***bta-miR-192***	1,060,828	***bta-miR-192***	1,272,798
2	***bta-miR-143***	613,476	***bta-miR-143***	778,165	***bta-miR-143***	961,386
3	***bta-miR-148a***	479,349	***bta-miR-148a***	535,207	***bta-miR-148a***	656,136
4	***bta-miR-26a***	177,987	***bta-miR-26a***	225,551	***bta-miR-26a***	273,009
5	***bta-miR-30a-5p***	163,172	***bta-miR-30a-5p***	180,528	***bta-miR-30a-5p***	225,973
6	***bta-miR-22-3p***	145,620	***bta-miR-22-3p***	156,016	***bta-miR-22-3p***	183,337
7	***bta-miR-27b***	110,066	***bta-miR-27b***	120,648	***bta-miR-27b***	154,701
8	***bta-let-7f***	108,663	***bta-let-7f***	117,980	***bta-let-7f***	126,847
9	***bta-miR-27a-3p***	70,791	***bta-miR-27a-3p***	77,866	***bta-miR-27a-3p***	96,651
10	***bta-miR-101***	65,614	***bta-miR-101***	74,408	***bta-miR-101***	90,249
11	***bta-miR-126-5p***	56,376	***bta-miR-21-5p***	66,516	***bta-miR-126-5p***	84,323
12	***bta-miR-21-5p***	52,293	***bta-miR-126-5p***	66,357	***bta-miR-21-5p***	73,380
13	***bta-miR-92a***	44,493	***bta-miR-191***	51,924	***bta-miR-191***	69,342
14	***bta-miR-191***	42,488	***bta-miR-215***	49,126	***bta-miR-92a***	60,130
15	***bta-let-7a-5p***	38,480	***bta-miR-92a***	47,179	*bta-miR-100*	53,849
16	***bta-miR-215***	35,905	***bta-let-7a-5p***	43,199	***bta-let-7a-5p***	50,790
17	*bta-miR-486*	30,488	*bta-miR-122*	37,749	***bta-miR-215***	46,908
18	*bta-miR-30e-5p*	30,000	*bta-miR-181a*	36,215	*bta-miR-122*	45,063
19	*bta-miR-100*	29,097	*bta-miR-26b*	33,505	*bta-miR-486*	42,147
20	*bta-miR-181a*	28,292	*bta-miR-30e-5p*	32,822	*bta-miR-26b*	41,433

Highly expressed miRNAs (n=16) which were commonly expressed across the three breed populations are highlighted in bold.

At a mirDeep2 score ≥ 4, an estimated probability that the predicted miRNA candidate is a true positive is greater than 70%, and with a significant Randfold p-value suggesting that the miRNA’s precursor sequence could be folded into a thermodynamically stable hairpin, we identified 126 (from 129 precursors), 101 (from 103 precursors), and 119 (from 125 precursors) novel miRNAs in Angus, Charolais, and KC samples, respectively. The identified novel miRNAs were largely expressed in one breed, with only 31 of them being commonly expressed across all the three breeds (Fig. 2b). Of the 241 unique novel miRNAs, *bta-miR-AB-10* and *bta-miR-AB-9* were the most expressed ones across the three breeds with an average of 52,817 and 46,932 reads aligned to these miRNAs, respectively (Table 2). The hairpin structure of the precursor and the read alignment distribution (i.e. alignment to the mature, star and loop sequences) of *bta-miR-AB-10* across the three breeds are presented in Fig. S1 in Supplementary File 1, indicating that the majority of the reads were aligned to mature micro RNA portion of the precursor. The top 20 expressed novel miRNAs and their miRDeep2 prediction scores are presented in Table 2 for each breed population, while all the identified novel miRNAs and their miRDeep2 prediction scores are provided in Supplementary File 3.

**Table 2:** Top 20 expressed novel miRNAs identified in the liver tissue of Angus, Charolais, and KC steers.

Provisional ID	miRDeep2 score	Estimated probability that the miRNA candidate is a true positive	Total read count	Mature miRNA consensus sequence
**Angus**				
*bta-miR-AB-10*	18,895.5	83 ± 5%	37,061	aaagcugaaugaacuuuuuggc
*bta-miR-AB-9*	4.9	77 ± 4%	35,497	agagaugaagcacuggagc
*bta-miR-AB-122*	5.5	83 ± 4%	9964	ugggcugcagugcgcuaugcc
*bta-miR-AB-83*	3438.3	83 ± 5%	6743	aaaaccugaaugaacuuuu
*bta-miR-AB-93*	1927.8	83 ± 5%	3784	aaagaaguuuguuuggguuuu
*bta-miR-AB-59*	5.1	83 ± 4%	3766	caaaaaguuuguuuggguuuu
*bta-miR-AB-65*	1854.7	83 ± 5%	3641	aaaaagguuuguuuggguuuu
*bta-miR-AB-27*	1789.4	83 ± 5%	3501	aaaaaaguuuguuuggauuuu
*bta-miR-AB-95*	5.2	83 ± 4%	3466	aaaaaaguuuguguggguuuu
*bta-miR-AB-52*	1663.5	83 ± 5%	3254	aaaaaaguuuguuugguuuuu
*bta-miR-AB-29*	1435.9	83 ± 5%	2816	acucgaacgaauuuuuggcc
*bta-miR-AB-3*	4.8	77 ± 4%	2725	guccaguuuucccaggaa
*bta-miR-AB-2*	6.2	84 ± 5%	1536	gggggccggcggcggcggcggc
*bta-miR-AB-54*	4.6	77 ± 4%	1210	gaaaaaguuuguuuggguuu
*bta-miR-AB-67*	4.3	77 ± 4%	1116	aaaaaaguuuguuugggauu
*bta-miR-AB-28*	4.8	77 ± 4%	1051	caaaaaguucguccagauuuu
*bta-miR-AB-12*	4.9	77 ± 4%	1041	aucccacuucugacacca
*bta-miR-AB-23*	502	83 ± 5%	985	acaaccugaaugaacuuuuuga
*bta-miR-AB-19*	5.1	83 ± 4%	976	ucaaguagcucacagucuag
*bta-miR-AB-63*	467.3	83 ± 5%	915	ggaauaccggguacuguaggcu
**Charolais**				
*bta-miR-AB-10*	29,069.9	77 ± 6%	57,018	aaagcugaaugaacuuuuuggc
*bta-miR-AB-9*	4.9	70 ± 4%	54,090	agagaugaagcacuggagc
*bta-miR-AB-3*	4.8	70 ± 4%	3570	guccaguuuucccaggaa
*bta-miR-AB-29*	1680.6	77 ± 6%	3296	acucgaacgaauuuuuggcc
*bta-miR-AB-19*	5.1	78 ± 5%	1897	ucaaguagcucacagucuag
*bta-miR-AB-2*	6.2	78 ± 5%	1291	gggggccggcggcggcggcggc
*bta-miR-AB-12*	4.9	70 ± 4%	1173	aucccacuucugacacca
*bta-miR-AB-23*	569.8	77 ± 6%	1118	acaaccugaaugaacuuuuuga
*bta-miR-AB-148*	561.9	77 ± 6%	1101	uuguccgacucuuagcgg
*bta-miR-AB-28*	4.8	70 ± 4%	1046	caaaaaguucguccagauuuu
*bta-miR-AB-137*	416.7	77 ± 6%	818	aaaucugaacaagcuuuuuggc
*bta-miR-AB-156*	406.5	77 ± 6%	796	aaaaaguucguuuggguuuuu
*bta-miR-AB-7*	401.1	77 ± 6%	785	aaaacugaaugaacauuuuggc
*bta-miR-AB-48*	333.4	77 ± 6%	653	cgaaaaguucguuuggguuuu
*bta-miR-AB-47*	251.3	77 ± 6%	491	aaaaguucguuucgguuuuucc
*bta-miR-AB-145*	4.3	70 ± 4%	381	ucuuggagcucaccgucuag
*bta-miR-AB-168*	4.7	70 ± 4%	375	cugaccuaugaauugaag
*bta-miR-AB-158*	190.1	77 ± 6%	372	aaaaaguuccuuuggguuuuc
*bta-miR-AB-34*	174.6	77 ± 6%	341	ucuagaagcucacagucuag
*bta-miR-AB-146*	171.8	77 ± 6%	335	uucucagguuggacaguccuga
**Kinsella composite (KC)**				
*bta-miR-AB-10*	32,819.1	80 ± 5%	64,372	aaagcugaaugaacuuuuuggc
*bta-miR-AB-9*	4.9	74 ± 4%	51,210	agagaugaagcacuggagc
*bta-miR-AB-225*	3118.6	80 ± 5%	6111	cucucgagucgcgacguguaucuc
*bta-miR-AB-59*	5.1	80 ± 4%	5151	caaaaaguuuguuuggguuuu
*bta-miR-AB-65*	2566.5	80 ± 5%	5037	aaaaagguuuguuuggguuuu
*bta-miR-AB-206*	5.2	80 ± 4%	5028	gaaaaaguuuguuuggguuuu
*bta-miR-AB-27*	5.3	80 ± 4%	4809	aaaaaaguuuguuuggauuuu
*bta-miR-AB-3*	4.8	74 ± 4%	4745	guccaguuuucccaggaa
*bta-miR-AB-52*	2256.4	80 ± 5%	4417	aaaaaaguuuguuugguuuuu
*bta-miR-AB-29*	2249.6	80 ± 5%	4412	acucgaacgaauuuuuggcc
*bta-miR-AB-63*	1079.1	80 ± 5%	2115	ggaauaccggguacuguaggcu
*bta-miR-AB-54*	4.6	74 ± 4%	1591	gaaaaaguuuguuuggguuu
*bta-miR-AB-28*	4.8	74 ± 4%	1502	caaaaaguucguccagauuuu
*bta-miR-AB-23*	750.3	80 ± 5%	1472	acaaccugaaugaacuuuuuga
*bta-miR-AB-67*	4.3	74 ± 4%	1434	aaaaaaguuuguuugggauu
*bta-miR-AB-12*	4.9	74 ± 4%	1429	aucccacuucugacacca
*bta-miR-AB-2*	6.2	80 ± 5%	1320	gggggccggcggcggcggcggc
*bta-miR-AB-18*	406.1	80 ± 5%	790	aaacccugaaggaacuuuu
*bta-miR-AB-7*	376.6	80 ± 5%	737	aaaacugaaugaacauuuuggc
*bta-miR-AB-198*	289.8	80 ± 5%	567	aaaaucugaacaaacuuuu

### miRNA differential expression

**Figure 3:**
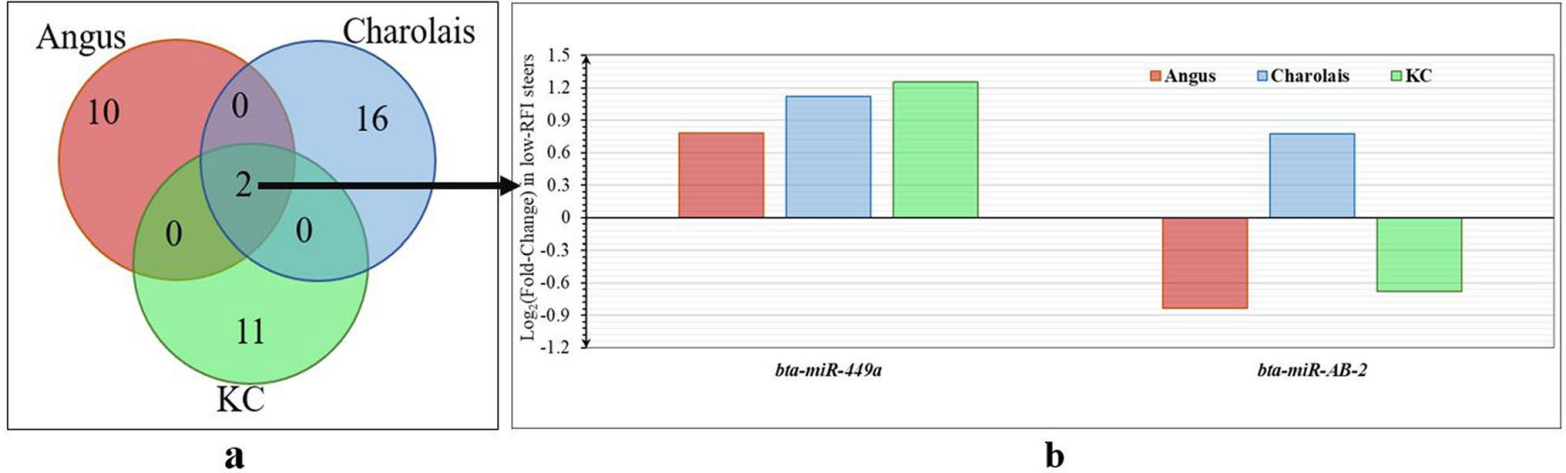
(**a**) Venn diagram showing differentially expressed miRNA overlaps among the studied populations [Angus, Charolais, and Kinsella Composite (KC)]; (**b**) Bar plot showing expression of *bta-miR-449a* and *bta-miR-AB-2* in low-RFI steers of the three breed populations.

Differential miRNA expression analysis was performed between the six (n = 6) low- and six (n = 6) high-RFI steer groups from the profiled animals for each breed population as selection of these 6 low- and 6 high-RFI resulted in an increased divergence between the low and high RFI animal groups. The low- and high-RFI steer groups of the three breed populations were all significantly different in their average RFI phenotypic values at P-value < 0.0042 after the Bonferroni correction for multiple comparisons (Table S2 in Supplementary File 1). For other traits, low-RFI animals consumed significantly less feed per day than their high-RFI counterparts in Charolais and KC. In Angus, low-RFI animals on average also consumed less feed as compared to the high-RFI animals although the difference did not reach the level of statistical significance (i.e. P-value > 0.0042). The average phenotypic values of other traits including animal slaughter age were not significantly different between the high and low RFI groups (Table S1 in Supplementary File 1). At a fold change (FC) > 1.5 and a P-value < 0.05, we identified 12 DE miRNAs in the liver tissue of Angus including 10 known miRNAs and two novel miRNAs (Table 3). Of these DE miRNAs, five were downregulated and seven were upregulated in low-RFI animals. In Charolais, we identified 18 DE miRNAs including 16 known miRNAs and two novel miRNAs, of which six were downregulated and 12 were upregulated in low-RFI Charolais steers (Table 3). In KC, 13 DE miRNAs including 10 known miRNAs and three novel miRNAs were identified, with five downregulated and eight upregulated in low-RFI steers (Table 3). Of all the identified DE miRNAs, *bta-miR-449a and bta-miRNA-2* were common to all three breeds (Fig. 3). Of the two common DE miRNAs, bta-miR-449a was upregulated in the low-RFI steers of all three breed populations whereas *bta-miRNA-AB-2* was upregulated in Charolais, and downregulated in both Angus and KC low-RFI steers (Fig. 3).

**Table 3:** Differentially expressed known and novel micro RNAs (miRNAs) between high and low-RFI animals within each breed population [Angus, Charolais, and Kinsella Composite (KC)], with a differential expression threshold of P-value < 0.05 and Fold-change (FC)  > 1.5.

Angus	MicroRNA	logCPM	LR	logFC	P-value
Known	***bta-miR-11985***	**1.621**	**13.815**	**− 1.377**	2.02E−04**
	*bta-miR-2285bg*	1.137	8.352	1.185	3.85E−03
	*bta-miR-2285n*	1.387	8.151	1.082	4.30E−03
	*bta-miR-2285u*	2.058	7.091	0.836	7.75E−03
	*bta-miR-424-3p*	1.518	6.951	0.959	8.38E−03
	*bta-miR-27a-5p*	1.221	5.840	− 0.978	0.016
	*bta-miR-24*	1.242	5.788	− 0.967	0.016
	*bta-miR-507b*	1.399	5.536	− 0.9	0.019
	*bta-miR-449a*	1.701	5.152	0.782	0.023
	*bta-miR-133b*	2.064	4.528	0.663	0.033
Novel	***bta-miR-AB-2***	**13.325**	**14.568**	− **0.833**	**1.35E**−04**
	*bta-miR-AB-47*	12.173	6.063	0.617	0.014
**Charolais**					
Known	***bta-mir-2415-3p***	**3.608**	**29.032**	**1.261**	**7.12E**−08**
	***bta-mir-2419-5p***	**6.405**	**24.192**	**0.797**	**8.72E**−07**
	*bta-mir-2285i*	1.906	9.603	1.117	0.002
	*bta-mir-133a*	5.218	9.457	− 0.966	0.002
	*bta-mir-449a*	1.456	9.648	0.64	0.002
	*bta-mir-2346*	0.871	8.829	− 1.33	0.003
	*bta-mir-1842*	1.058	6.220	− 1.026	0.013
	*bta-mir-2284ac*	2.414	6.068	0.656	0.014
	*bta-mir-2285ai-5p*	2.442	5.946	0.645	0.015
	*bta-mir-12001*	1.884	5.527	− 0.729	0.019
	*bta-mir-299*	1.565	5.103	0.772	0.024
	*bta-mir-2284c*	0.490	4.773	− 1.104	0.029
	*bta-mir-485*	0.816	3.860	0.747	0.043
	*bta-mir-6521*	1.445	3.855	− 0.872	0.049
	*bta-mir-365-5p*	1.320	4.076	0.916	0.049
	*bta-mir-7859*	0.659	3.860	0.696	0.0496
Novel	***bta-miR-AB-2***	**13.410**	**10.823**	**0.77**	0.001**
	*bta-miR-AB-15*	11.255	5.830	0.735	0.0158
**KC**					
Known	***bta-miR-190a***	**1.873**	**77.302**	− **3.133**	**1.47E**−18**
	***bta-miR-449a***	**2.225**	**20.035**	**1.252**	**7.61E**−06**
	***bta-miR-155***	**6.653**	**17.100**	**0.829**	**3.55E**−05**
	***bta-miR-424-5p***	**4.561**	**12.718**	**0.756**	**3.62E**−04**
	***bta-miR-223***	**5.207**	**12.131**	**0.737**	**4.96E**−04**
	*bta-miR-1246*	4.554	10.385	− 0.683	1.27E−03
	*bta-miR-363*	1.947	8.661	0.866	0.003
	*bta-miR-147*	2.006	7.647	0.801	0.006
	*bta-miR-2411-3p*	1.834	5.032	− 0.677	0.025
	*bta-miR-2483-5p*	0.672	4.255	0.877	0.039
Novel	***bta-miR-AB-63***	**16.176**	**77.900**	− **0.864**	**9.58E**−04**
	*bta-miR-AB-225*	14.839	5.533	1.228	0.002
	*bta-miR-AB-2*	11.531	4.147	− 0.677	0.013

The sign of logFC shows the direction of miRNA expression in low-RFI steers relative to high-RFI animals. The miRNAs that were significantly differentially expressed at a more stringent threshold of Bonferroni false discovery rate (FDR) correction < 0.05 and Fold-change > 1.5 are highlighted in bold and a “**” was added to their P-values. logCPM = log_2_ (counts per million), *LR* = likelihood ratio test static value, and **logFC** = log_2_(Fold-change).

Additionally, at a more stringent false discovery rate (FDR) < 0.05 (Bonferroni correction for multiple tests) and a FC > 1.5, two (*bta-miR-11985 and bta-miR-AB-2*), three (*bta-mir-2415-3p*, *bta-mir-2419-5p,* and *bta-miR-AB-2*) and six (*bta-miR-190a, bta-miR-449a*, *bta-miR-155, bta-miR-424-5p, bta-miR-223,* and *bta-miR-AB-63*) DE miRNAs were identified in Angus, Charolais, and KC, respectively, as highlighted in Table 3.

### Target gene prediction and functional enrichment analyses for the most abundant known and novel miRNAs

We performed target gene prediction for 18 miRNAs (16 known and 2 novel miRNAs *bta-miR-AB-10 and bta-miR-AB-9*) that were identified as the most expressed across the three breed populations for functional enrichment analyses (Tables 1, 2). At a threshold cut off of not less than the 99th context++ score percentile, we identified 1022 target genes. A list of all these identified targets, their respective TargetScan scores for the fourteen parameters, and the mRNA-miRNA correlation coefficients in the samples of the three populations are provided in Supplementary File 4. Of these target genes, 1008 mapped to the IPA ingenuity database, and they are mainly involved in cell morphology, cellular assembly and organization, cellular growth and proliferation, free radical scavenging, and cell death and survival biological functions. All metabolic and cellular functions significantly enriched by the identified targets are presented in Fig. S2 of Supplementary File 1. For cell morphology, the target genes are mainly involved in maintaining morphology of cellular organelles such as nucleus and cellular the transmembrane potential. For cellular growth and proliferation, the target genes are involved in proliferation of liver cells, and proliferation and development of different immune cells such as macrophages, lymphocytes, and natural killer cells, whereas for cell death and survival biological function, the target genes are primarily involved in necrosis and apoptosis. Also, IPA identified sirtuin signaling, 1L-12 signaling and production in macrophages, tryptophan degradation III, receptor recognition of bacteria and viruses and senescence pathway, as presented in Fig. S3 in Supplementary File 1.

### DE miRNAs target gene prediction

For the differentially expressed known and novel miRNAs within each breed (Table 3), we identified 767, 1667, and 787 target genes for Angus, Charolais, and KC, respectively, at a minimum context++ score percentile of 99. The targets, their targeting DE miRNAs, the target-miRNA TargetScan estimated scores and the mRNA-miRNA correlation coefficients in the studied animals from each breed are presented in Supplementary File 5. Functional enrichment analysis for the target genes identified for DE miRNAs in Angus are mainly involved in regulating the gene expression of cell cycle, gene expression modulation, cellular assembly and organization, DNA replication, recombination and repair and RNA post-transcription modification (Fig. S4 in Supplementary File 1). Additionally, the major pathways enriched by these target genes included HIPPO signaling, GADD45 signaling, ATM signaling, endoplasmic reticulum stress pathway, and Granulocyte adhesion and diapedesis pathway as shown in Fig. S5 in Supplementary File 1. For Charolais, the identified targets are majorly involved in protein synthesis, protein trafficking, RNA post-translation modification, lipid metabolism, and molecular transport (Fig. S6 in Supplementary File 1). Estrogen receptor signaling, granzyme A signaling, sirtuin signaling pathway, protein ubiquitination pathway, and coagulation system pathway were identified as among the major canonical pathways enriched by Charolais DE miRNA target genes (Fig. S7 in Supplementary File 1). For KC, cell signaling, RNA post-translation modification, protein synthesis, cell-to-cell signaling and interaction, cellular growth and proliferation were identified as the major molecular and cellular functions enriched by the targets genes of the DE miRNAs identified (Fig. S8 in Supplementary File 1). Additionally, RhoGDI signaling, Ras homolog family member A (RhoA) signaling, signaling by Rho family GTPases, oxidative phosphorylation and diphthamide biosynthesis were identified as the major pathways enriched by the target genes (Fig. S9 in Supplementary File 1).

### DE miRNAs target gene prediction among DE genes previously identified

**Figure 4:**
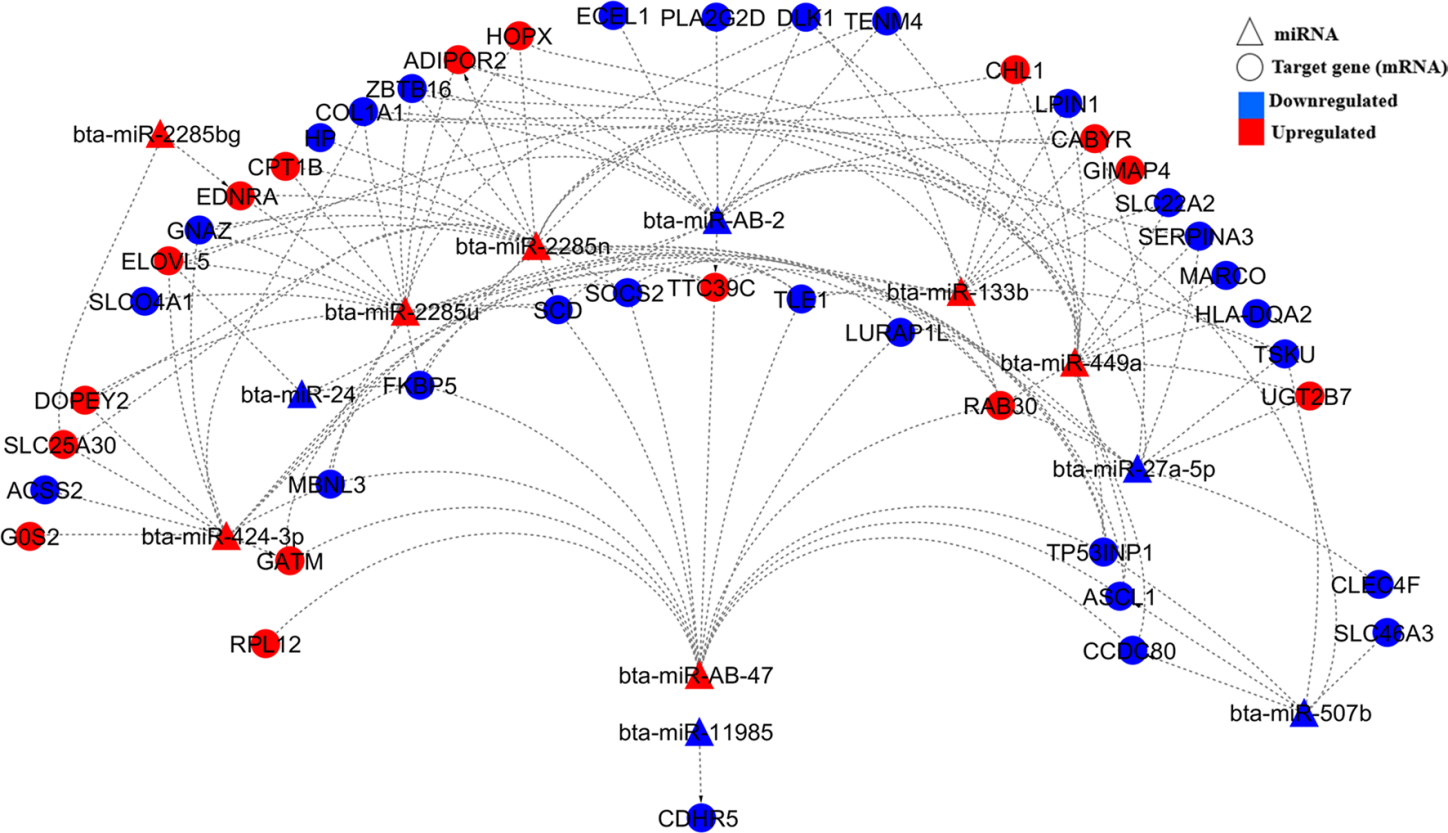
DE target genes and DE miRNAs interaction network generated using Cytoscape and regulation of both DE miRNAs and DE target genes in the liver tissue of low-RFI Angus steers.

**Figure 5:**
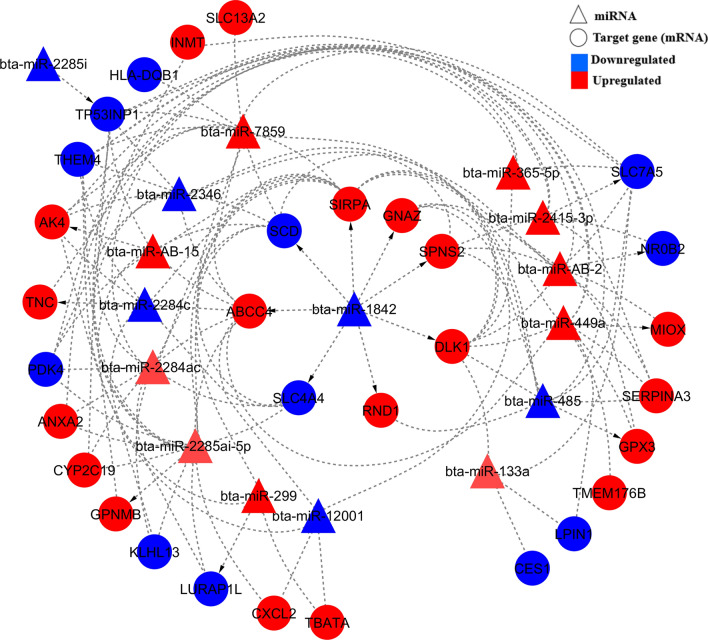
DE target genes and DE miRNAs interaction network generated using Cytoscape and regulation of both DE miRNAs and DE target genes in the liver tissue of low-RFI Charolais steers.

**Figure 6:**
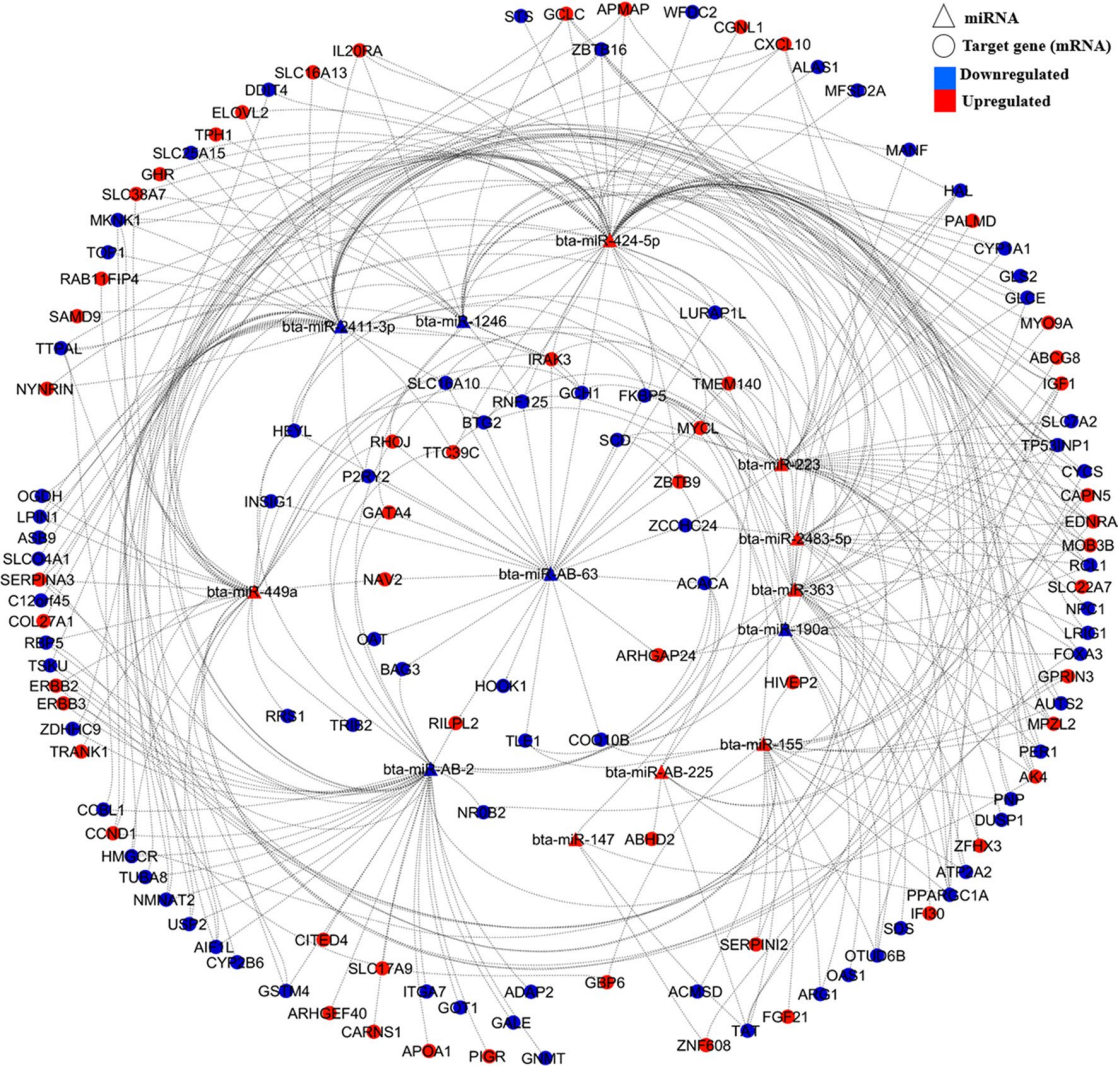
DE target genes and DE miRNAs interaction network generated using Cytoscape and regulation of both DE miRNAs and DE target genes in the liver tissue of low-RFI Kinsella Composite (KC) steers.

Within the same breed populations used in the current study, we previously identified 72, 41, and 175 DE genes between low- and high-RFI steer groups for Angus, Charolais, and KC, respectively^18^. At a minimum context++ score percentile of 99, one (*GNAZ*), two (*THEM4* and *CES1*), and five (*PALMD*, *C12orf45*, *IRAK3*, *CITED4*, and *IL20RA*) out of the 72, 41, and 175 DE genes were predicted as targeted DE genes for the DE miRNAs, respectively, in the same Angus, Charolais, and KC populations. However, at a lower threshold (> 50th context++ score percentile), we detected 44, 31, and 129 target DE genes out of the 72, 41, and 175 DE genes as potential targets for the DE miRNAs identified in the current study for Angus, Charolais, and KC animals, respectively. All DE genes (as reported by Mukiibi et al.^18^) identified as targets of the DE miRNAs in the current study and their mRNA-miRNA correlation coefficients for all the three breeds are presented in Supplementary File 5 at a minimum context++ score percentile of 99 and in Supplementary File 6 at a threshold > 50th context++ score percentile. These indicated that approximately 70% of the DE genes were potentially targeted by the identified miRNAs in this study, ranging from 61.1% for Angus to 75.6% for Charolais. Of the 44, 31, and 129 target DE genes, 31, 24, and 89 genes in Angus, Charolais, and KC, respectively, were predicted to be targeted by multiple DE miRNAs. However, a few of the DE target genes were predicted to be targeted by a single miRNA, and it was observed that the up-regulation DE miRNAs did not always lead to down-regulation of DE genes in the liver tissue of low-RFI animals as demonstrated in Figs. 4, 5 and 6 for Angus, Charolais, and KC, respectively. Also, most of the DE miRNAs were predicted to predominantly target multiple genes for Angus, Charolais, and KC (Figs. 4, 5, and 6). The miRNA *bta-miR-449a,* which was identified as a common and upregulated DE in low-RFI steers of all the three breeds, was predicted to target 16, 11, and 35 DE genes in Angus, Charolais, and KC steers, respectively, of which only three target genes including (*SERPINA3*, *TP53INP1* and *LPIN1*) were common to all the three breeds. Of the 16 target genes of *bta-miR-449a* identified in Angus, 12 and 4 were downregulated and upregulated respectively in the liver tissue of low-RFI animals (Fig. 4). Of the 11 targets identified for *bta-miR-449a* in Charolais, 4 and 7 were downregulated and upregulated respectively in the liver tissue of low-RFI animals (Fig. 5). In KC, of the 35 targets by *bta-miR-449a* 23 and 12 were downregulated and upregulated, respectively, in the liver tissue of low-RFI animals (Fig. 6).

In Angus, *RAB30* and *FKBP5* genes were found to be the major target genes for the identified DE miRNAs*,* with each predicted to be targeted by six miRNAs (Fig. 4). The other six major target genes including *COL1A1, ELOVL5, SCD, TLE1, TP53INP1,* and *TTC39C,* were predicted to be regulated by five DE miRNAs each, as shown in Fig. 4*.* Of these targets, *FKBP5*, *COL1A1*, *SCD*, *TLE1,* and *TP53INP1* were downregulated in low-RFI animals, whereas *RAB30*, *ELOVL5,* and *TTC39C* were upregulated in the same animals. Additionally*,* the miRNAs and DE target gene interaction networks showed *bta-miR-2285n, bta-miR-2285u,* and *bta-miR-449a* (all upregulated) as the major miRNAs, targeting 23, 19, and 16 DE genes, respectively (Fig. 4). Other major miRNAs including *bta-miR-AB-2, bta-miR-AB-47*, and *bta-miR-424-3p*, are predicted to regulate 14, 13, and 12 DE genes, respectively. We also identified that 39 DE genes were targeted by one or multiple upregulated (in low-RFI animals) DE miRNAs in Angus, of which 23 were downregulated in low-RFI animals whereas 16 were upregulated in the animals of low-RFI.

For Charolais, the major targeted genes included *SIRPA* (predicted to be targeted by 10 DE miRNAs), *ABCC4* (predicted to be targeted by nine DE miRNAs), *DLK1,* and *TP53INP1* (each predicted to be targeted by eight DE miRNAs), *SCD*, *SLC7A5,* and *THEM4* (each predicted to be targeted by seven DE miRNAs), and *AK4* (predicted to be regulated by six DE miRNAs) (Fig. 5). *SLC7A5, TP53INP1, SCD, and THEM4* were downregulated*,* whereas *AK4, SIRPA, DLK1, and ABCC4* were upregulated in low-RFI animals*.* Furthermore, *bta-miR-2285ai-5p* (14 targets), *bta-miR-7859* (12 targets), *bta-miR-2284ac* (11 targets), *bta-miR-AB-2* (11 targets), *bta-miR-449a* (10 targets) were predicted to be major regulators among the identified DE miRNAs and were all upregulated in liver tissue of low-RFI steers, as shown in Fig. 5. *SIRPA *(upregulated in low-RFI steers) was predicted to be co-regulated by all the five major DE miRNAs. Thirteen of the 30 DE genes predicted to be targeted by either single or multiple upregulated (in low-RFI steers) DE miRNAs were downregulated, whereas the other 17 were upregulated (low-RFI steers) even though they were predicted to be targeted by multiple upregulated DE miRNAs in Charolais.

For KC, the main predicted target DE genes included *FKBP5* (targeted by 10 DE miRNAs), *TP53INP1* (targeted by 8 DE miRNAs), *PPARGC1A* and *IGF1* (both targeted by 7 DE miRNAs), *EDNRA*, *GCH1*, *MKNK1*, *IRAK3,* and *HEYL,* which were targeted by 6 DE miRNAs each. *HEYL, FKBP5, TP53INP1, PPARGC1A, MKNK1,* and *GCH1*were downregulated in the liver tissue of low-RFI animals, whereas *IGF1, IRAK3* and *EDNRA* were upregulated in the liver tissue of the same animals. Among the DE miRNAs, *bta-miR-424-5p* (targeting 61 DE genes), *bta-miR-AB-2 *(targeting *41* DE genes)* bta-miR-2411-3p* (targeting 40 DE genes), bta-miR-223 (targeting 36 DE genes), and *bta-miR-449a* (targeting 35 DE genes) were identified as major regulators, as shown in Fig. 6. Other main DE miRNAs included *bta-miR-AB-63* (targeting 28 DE genes), *bta-miR-363* (targeting 26 DE genes), *bta-miR-155* (targeting 23 DE genes), *bta-miR-1246* (targeting 20 DE genes), and *bta-miR-2483-5p* (targeting 19 DE genes) (Fig. 6). Of the 106 DE genes predicted to be targeted by a single or multiple upregulated (in low-RFI steers) DE miRNAs, 62 were upregulated in the liver tissue of the same animals. However, the other 44 DE genes were upregulated despite being targeted by either a single or multiple upregulated miRNAs (Fig. 6).

Functional enrichment analysis of the DE miRNA targeted DE genes identified multiple biological functions that greatly overlapped with those identified for all the DE genes reported by Mukiibi et al.^18^. For Angus, the DE miRNA target DE genes are mainly involved in lipid metabolism, molecular transport, small molecule biochemistry, energy production, and carbohydrate metabolism, as shown in Fig. S10 in Supplementary File 1. For Charolais, lipid metabolism, molecular transport, small molecule biochemistry, cellular movement, and cell-to-cell signaling and interaction were identified as the main biological functions enriched by the identified DE targets, as shown in Fig. S11 in Supplementary File 1. For KC, the DE genes targeted by the DE miRNA are primarily involved in amino acid metabolism, small molecule biochemistry, lipid metabolism, vitamin and mineral metabolism, and cell death and survival, as presented in Fig. S12 in Supplementary File 1. All the target DE genes involved in each of the top five significantly enriched cellular and metabolic functions for the three breeds are presented in Table S3 in Supplementary File 1.

## Discussion

RNA sequencing offers a greater resolution to profile miRNAs, even at very low levels of expression in the cells^31^, and allows for the parallel profiling of abundance of known miRNAs as well as the identification of novel miRNAs^32^. Additionally, the availability of miRNA sequences enables the prediction of potential target genes for both known and novel miRNAs^31^. In the current study we employed deep sequencing of small RNAs to profile miRNA expression in the liver tissues of 60 beef steers from three distinct beef breed populations. We obtained high quality sequence data as revealed by our sequencing quality results, with an average Phred quality score of 37.3 across the samples of the three breeds. Additionally, sequence data quality control processing (including removal of other small miRNAs) resulted in read sequences with an average length of 21 bp, and the majority of the reads ranged between 20 and 24 bp across the samples from the three breeds, as shown in Fig. 1a, providing high quality and reliable reads for downstream alignment and abundancy profiling of miRNAs^33^. Furthermore, to validate the RNAseq miRNA profiling results, we conducted TaqMan qPCR differential expression analysis on six selected miRNAs (Table S4 in Supplementary File 1). It showed that all expression levels in low-RFI animals were consistent between the RNAseq and TaqMan qPCR (Fig. S13 and Table S4 in Supplementary File 1), with the P-values of significance tests of qPCR between high and low-RFI animals groups ranging from a significant P-value (< 0.05) for *bta-miR-133a* (P-value = 0.003) to suggestive P-values (P-value = 0.054 and P-value = 0.086) for *bta-miR-223* and *bta-miR-424-5p,* respectively, to relatively higher P-values ranging from 0.144 to 0.314 for the other three miRNAs, as shown in Fig. S13 and Table S4 in Supplementary File 1. Although the differential expression P-values for qPCR results were not as low as those for RNAseq, which might be related to sensitivity differences between the two methods regarding profiling gene expression^31^, we observed that the Pearson correlation coefficients were high (0.73 – 0.99) between the qPCR and RNAseq profiles and significant (6.79E−03 to 6.00E−10) (Table S5 in the Supplementary File 1), which indicates that the RNAseq results were highly reproducible.

With a high average alignment rate close to 75%, we found that approximately 90% of the identified miRNAs were expressed in all the three breed populations, indicating a high level of similarity among the breeds in terms of hepatic miRNA expression. Similarly, high similarity rates in expression patterns were also observed for the protein coding genes in the same populations, where over 96% of the expressed genes were commonly expressed among the liver tissues of the same beef cattle populations^18^. Of the expressed miRNAs, 10 were highly expressed including *bta-miR-192, bta-miR-143, bta-miR-148a, bta-miR-26a, bta-miR-30a-5p, bta-miR-22-3p, bta-miR-27b, bta-let-7f, bta-miR-27a-3p,* and *bta-miR-101*, and these accounted for an average of 78.2% of the aligned reads for the profiled liver tissues of the studied animals across the three breeds (Table 1). The miRNAs *bta-miR-101, bta-miR-143, bta-miR-30a-5p, bta-let-7f, bta-miR-192,* and *bta-miR-148a* were previously reported among the 10 top most expressed miRNAs in the liver tissue of Australian Angus steers^28^ and Chinese Holstein dairy cows^34^, indicating their stable high expression across a wide range of cattle breeds, despite the genetic distinctiveness of these animals. Additionally, Sun et al. reported high expression levels of these miRNAs in multiple tissues, with *bta-miR-143* and *bta-miR-27b* particularly showing high levels of expression in all the tissues from both beef and dairy animals^34^. High expression of these miRNAs in different tissues indicates their potentially critical importance to modulate key biological functions in those bovine tissues. The miRNA *bta-miR-192,* which was the most expressed miRNA across the three breed populations, belongs to the *miR-192/215* family, whose homologous members have been implicated in several biological functions and disease disorders in different species. For example, *miR-192* has been reported in mice to regulate genes involved in glucose metabolism, cell adhesion and migration, tumorigenesis and tumor progression, protein SUMOylation, epigenetic regulation and epithelial-mesenchymal transition of the hepatic cells through the *HNF4*-*miR-194*/ *miR-192* signaling pathway^35^. In sheep, *miR-192* has been reported to be involved in regulating the growth and development of skeletal muscle^36^.

To explore the biological importance of the highly expressed miRNAs across the liver tissues of the studied animals, we performed target gene prediction of 18 miRNAs that were highly expressed in steers from the three breeds. Functional enrichment analysis of the target genes revealed that the candidate target genes were involved in key biological processes including maintaining cellular homeostasis, immune functions, proliferation of liver cells, and apoptosis of cells. Consistent with our results, some of the most abundant miRNAs have been identified as important modulators of liver cellular metabolic homeostasis, liver cell proliferation and development, liver cell death and regeneration in different species^26^. For example, *miR-143* which was the second most abundant miRNA in our studied samples was reported to be involved in glucose and insulin metabolism in mice^37^. *miR-148* and *miR-26a* are involved in the regulation of mice hepatocyte proliferation^38,39^, a key process in liver tissue regeneration. In human and mouse liver tissue, *miR-148a*^40^ and miR-27^41^, respectively, have been identified as regulators of liver detoxification. Based on our results and the conserved nature of the miRNA-mRNA interactions across mammalian species^24^, we speculate that these highly expressed miRNAs in the bovine liver might play similar functions as those highlighted in other species. Furthermore, the involvement of these miRNAs in proliferation of different cells as well as cell death and survival is plausible, since the liver is in a constant state of self-regeneration or regrowth to recover hepatic tissue lost due to assault by pathogens, toxins, and exogenous antigens^42,43^. Liver regeneration is a complex and highly regulated process that includes the initiation phase, the cell proliferation phase, and the regeneration termination phase, all of which are likely modulated by miRNAs^44,45^. However, further molecular studies beyond Insilco predictions are needed to precisely validate the target genes of these miRNAs and their respective functions in cattle given the physiological differences between the species.

The liver is a central metabolic organ serving major biological functions in the mammalian body including nutrient (lipids, carbohydrates, proteins/amino acid, and vitamins and minerals) metabolism, xenobiotics and toxin metabolism, in addition to pathogen processing and growth regulation^46,47^. MicroRNAs are known to modulate all these functions^26^. Therefore, differential hepatic miRNA expression between efficient and inefficient animals can potentially contribute to the molecular variability for feed efficiency in beef cattle. In the current study we identified 39 differentially expressed (DE) known and novel miRNAs between the efficient and inefficient steer groups in all the three studied populations. However, most of the identified DE miRNAs (94.9%) were breed specific. These findings were in line with the results in our mRNA differential expression studies^18,27^ that involved the same animals, where most of the DE genes (from 63.4% for Angus to 84.6% for KC) associated with RFI^18^ and its component traits^27^ were breed specific as well, which we speculate may be due to genetic uniqueness of the studied breeds. However, since we used a lower threshold or raw P-value < 0.05 to identify the differentially expressed miRNAs, further studies involving larger beef cattle populations and more divergent phenotype animal groups would be required to validate the current results.

Within each breed, most (i.e. 58.3% for Angus, 66.7% for Charolais, and 61.5% for KC) of the differentially expressed miRNAs were upregulated in low-RFI animals, and hence suggests a general expectation of reduced expression of their target genes. Compared with previous studies that have investigated the association of miRNA expression with feed efficiency^28,29^, *bta-miR-424-5p* was the only miRNA that was commonly DE in our study and in the Austrian Angus population^28^. However, in our study, *bta-miR-424-5p* was upregulated in the liver tissue of low-RFI animals (KC), whereas it was identified as downregulated in the liver tissue of low-RFI Angus bulls^28^. This difference could be due to genetic, physiological or environmental differences between the animals studied in the two studies. It is also worth noting that Al-Hussein et al*.* sequenced two cDNA libraries of pooled RNA from efficient and inefficient bulls^28^, whereas in our study we sequenced each individual cDNA library for each of the 60 studied steers, and this could also contribute to the differences between the two studies.

To investigate the potential biological importance of the DE miRNAs related to RFI within each breed, we predicted their target genes at a TargetScan context++ score percentile threshold of 99 for each breed. The key biological functions associated with the target genes included cell cycle, cellular growth and proliferation, cellular assembly and organization, lipid metabolism, protein synthesis, protein trafficking, cell-to-cell signaling and interaction, molecular transport, post-translational modification, and RNA post-transcription and translation modification. Of these functions, cell signaling, cellular growth and proliferation, lipid metabolism, protein synthesis, cellular development, cell death and survival molecular transport and protein synthesis have been previously reported to be associated with feed efficiency in beef cattle through liver transcriptomic studies^10,11,15,18^. To further investigate the interactions between the identified DE miRNAs and the 72, 41, and 175 differentially expressed (DE) genes that were previously identified in the same liver tissues of the same three animal populations^18^, we identified only 1 (*GNAZ*), 2 (*THEM4* and *CES1*), and 5 (*PALMD*, *C12orf45*, *IRAK3*, *CITED4*, and *IL20RA*) out of the 72, 41, and 175 DE genes as targeted DE genes for the DE miRNAs, respectively, in the Angus, Charolais, and KC breed populations using a TargetScan context++ score percentile threshold of 99 for each breed populaton. The low number of targeted DE genes by the DE miRNAs for each breed population would not allow for meaningful gene network and functional analysis. Thereore, we further used a lower threshold of context++ score percentile > 50 to predict targeted DE genes out of the 72, 41, and 175 DE genes of each breed population since a context++ score percentile > 50 is also considered as a high context score to shows that a specific site is more favorable than most other sites of the miRNA for target gene identifiction^48^. With a context++ score percentile > 50, it was revealed that 61.1% (44 DE genes), 75.6% (31 DE genes) and 73.7% of the previously identified DE genes were predicted as potential targets of the DE miRNAs in Angus, Charolais, and KC steers, respectively. These target DE genes are mainly involved in lipid metabolism, molecular transport, small molecule biochemistry, energy production, carbohydrate metabolism, cellular movement, cell-to-cell signaling and interaction, cell death and survival, amino acid metabolism and vitamin and mineral metabolism, implying that these identified DE miRNAs influence feed efficiency through differential modulation of these biological functions in the liver. From the DE miRNA–DE mRNA interaction networks in Figs. 4, 5 and 6, it can be observed that some miRNAs were predicted to target multiple DE genes, and some single genes were predicted to be targets for multiple miRNAs. These complex forms of miRNA–mRNA interactions emanate from the fact that a single miRNA using its seed region can bind to multiple sites in the 3′-UTRs of different genes (mRNAs), and also one target can have multiple binding sites for several miRNAs^23,49^, hence allowing miRNAs to modulate multiple biological processes even though they are small in number compared with the mRNAs that they regulate.

To a large extent, we observed contrasting expression patterns in DE miRNAs compared with DE genes in the liver tissues as expected since the main mode of action of miRNAs is through promoting deadenylation of their target transcripts which conquently results into accelerated degradation of those target mRNAs^50^. As an example, of the identified DE target genes for the upregulated miRNAs *bta-miR-2285n*, *bta-miR-2285u*, *bta-miR-449a* and *bta-miR-47* in Angus low-RFI animals, 52%, 63%, 75% and 62% were downregulated, respectively. For Charolais, of the predicted DE targets of upregulated miRNAs *bta-miR-2285ai-5p*, *bta-miR-7859*, *bta-miR-2284ac* and *bta-miR-449a* in low-RFI animals, nearly 50%, 50%, 64% and 36%, respectively, were downregulated. In KC, of the predicted DE targets of upregulated DE miRNAs *bta-miR-424-5p*, *bta-miR-223*, *bta-miR-449a,* and *bta-miR-363* in low RFI animals, approximately 62%, 67%, 66% and 54%, respectively, were downregulated. However, we also observed a significant number of upregulated predicted target genes despite being targeted by multiple upregulated miRNAs. These observations could be attributed to the different mechanisms of miRNA gene regulation in mammalian cells including the augmentation of mRNA degradation through deadenylation and translation (proteins) repression when the miRNAs bind to the 3′ UTRs of their targets^50^. Therefore, the observation that some DE genes predicted to be targeted by upregulated DE miRNAs were of downregulated in the liver tissue of low-RFI animals could due to the augmented mRNA degradation mode of regulation. On the other hand, protein translation repression could be the main miRNA gene expression modulation mode for those genes that retained high expression profiles despite being targeted by multiple upregulated DE miRNAs. To better understand the relationship of miRNA and mRNA, the correlation coefficients between miRNA and mRNA of the target genes were also investigated. In general the negative correlation coefficients between the DE miRNAs and their DE target genes largely confirmed the DE miNRA-target mRNA regulatory interaction patterns in Figs. 4, 5, and 6, while the magnitudes of correlation coefficients shed some light on strength of their DE miNRA-target mRNA regulatory interactions. For Angus, miRNA *bta-miR-2285n* has the strongest negative correlation with the target gene *SCD* (r = − 0.49, Supplementary File 6), which indicates that increased expression of the miRNA would reduce *SCD* mRNA to a greater extent. Similar strong negative correlations were also found between *bta-miR-2346* and target gene *CYP2C19* for Charolais, and between *bta-miR-2483-5p* and target gene *ZBTB16* for KC. However, strong positive correlations were also detected between certain miRNA and target DE genes in the three breeds (Supplementary File 6), which suggests that these targeted genes are under different mechanisms of miRNA gene regulation to a greater extent. Although we used a context++ percentile score from greater than 50 to 100 as suggested by the Targetscan 7 documentation^48^ to predict targeted genes of miRNAs in this study, the interactions of the DE miNRA and target mRNAs should be interpreted with caution due to the nature of Insilco prediction that is subject to prediction accuracy. Hence, further molecular experiments are warranted to validate these interactions.

## Conclusions

In the current study we employed RNAseq to perform hepatic miRNAome profiling of beef steers from Angus, Charolais, and KC populations. We identified a total of 588 expressed known bovine miRNAs, of which 89.8% were commonly expressed in the liver tissue of the animals from the three populations. Of these miRNAs, *bta-miR-192, bta-miR-143, bta-miR-148a, bta-miR-26a, bta-miR-30a-5p, bta-miR-22-3p, bta-miR-27b, bta-let-7f., bta-miR-27a-3p* and *bta-miR-101* had the greatest expression levels in all three breeds. We also detected 241 novel bovine miRNAs expressed in the liver tissue, with 69.3% identified as expressed in only one of the three breeds whereas 12.9% were identified as expressed in all of the three populations. Differential miRNA expression analyses found 39 miRNAs that were associated with RFI, including five novel miRNAs (*bta-miR-AB-2, bta-miR-AB-47, bta-miR-AB-15, bta-miR-AB-63,* and *bta-miR-AB-225*). Most of the DE miRNAs were breed specific with only two miRNAs (*bta-miR-449a* and *bta-miR-AB-2*) being differentially expressed in all three breed populations. The predicted target genes of the identified DE miRNAs are involved in multiple cellular and molecular functions including cell cycle, cell death and survival, cell signaling, cellular growth and proliferation, lipid metabolism, protein synthesis, protein trafficking, cell morphology, cell-to-cell signaling and interaction, cellular development, molecular transport, post-translational modification, amino acid and carbohydrate metabolism. Additionally, the identified DE miRNAs were found to target approximately 70% of the previously identified DE genes in the liver tissue of feed efficient and inefficient animals from the same populations. These target DE genes are mainly involved in lipid metabolism, molecular transport, small molecule biochemistry, energy production, carbohydrate metabolism, cellular movement, cell-to-cell signaling and interaction, cell death and survival, amino acid metabolism, and vitamin and mineral metabolism. Our results provide further insight into hepatic miRNAome expression profiles of beef cattle and their potential molecular regulatory mechanisms relating to feed efficiency in beef cattle.

## Materials and methods

### Animal populations and management

The population description and management practices of the experimental animals used in the current study have been presented in our recent studies^18,27^. Briefly, the animals were raised and managed following the Canadian Council of Animal Care (CCAC) guidelines on the care and use of farm animals in research teaching and testing^51^, and all the experimental procedures applied to the animals were approved by the University of Alberta Animal Care and Use Committee (AUP00000777). The animal populations included a total of 256 beef steers from two purebred populations (i.e. Angus and Charolais), and one Kinsella Composite (KC) beef steer population. The steers were born, raised and managed similarly at the Roy Berg Kinsella Ranch, University of Alberta, Canada. All the purebred Angus and Charolais cows were serviced through estrous synchronization and artificial insemination, followed by natural service by purebred Angus or Charolais bulls whose pedigree records were maintained by the Canadian Angus or Charolais Association, respectively. The KC animals were produced through crossing Angus, Charolais, or Alberta Hybrid bulls with the University of Alberta’s hybrid dam line. The crossbreeding design used to generate the University of Alberta’s hybrid dam line was previously described by Goonewardene et al.^52^. Additionally, commercial crossbred bulls have been added to the KC herd since 2012 for natural service. The animals used in the current study were born between the months of April and May of 2014 and were castrated immediately after birth. The steer calves were maintained with their dams on pasture and weaned at an average age of about six months. The weaned animals were transitioned to a backgrounding diet composed of 80% barley silage, 17% barley grain, and 3% rumensin pellet supplement, and thereafter were fed set-up diets with gradually decreasing barley silage and increasing barley grain proportions for 3 weeks. Subsequently, the animals were introduced to the finishing diet of 75% barley grain, 20% barley silage, and 5% rumensin pellet supplement (as fed basis) after the transition period of 3 weeks. The nutrient composition of the finishing diet is provided in the Table S6 in the Supplementary File 1.

### GrowSafe feedlot test, phenotype measurement and RFI calculations

Between the months of April and August in 2015, 50 Angus, 48 Charolais, and 158 KC steers were measured for individual feed intake using the GrowSafe System (GrowSafe Systems Ltd., Airdrie, Alberta, Canada). A detailed description on measuring each individual animal’s daily feed intake using the GrowSafe automated system has been described by Mao et al.^53^ and also provided in our previous reports^18,27^. Briefly, the animals were tested for feed intake for a period ranging from 70 to 73 days, during which animals were fed on the finishing diet described above. Daily dry matter intake (DMI) of each animal was calculated as the average of the daily feed intake records over the test period and was standardized to 12 MJ ME per kg dry matter based on the energy content of the diet. Initial body weight (BW) and average daily gain (ADG) for each animal were obtained from a linear regression between serial body weight measurements and time (days) that were recorded on two consecutive days at the start of feedlot test, at approximately 14-day intervals during the feedlot test, and on two consecutive days prior to the end of the test. Metabolic mid-weight (MWT) was calculated as midpoint BW^0.75^, where midpoint BW was computed as the sum of the initial BW of the animal and the product of its ADG multiplied by half the number of days under the feedlot test. RFI was calculated as the difference between the actual standardized daily DMI and the expected DMI that was predicted using the regression intercept and regression coefficients of ADG and MWT on actual standardized daily DMI as proposed by Koch et al.^54^.

### Liver tissue collection

Tissue collection and processing procedures were previously described in our recent studies^18,27^. Briefly, all animals used in the current study were slaughtered at the Agriculture and Agri-Food Canada (AAFC) Lacombe Research Centre (Lacombe, AB) between July and September of 2015. Animals were considered ready for slaughter at an average back-fat thickness of 8 mm between the 12th and 13th ribs, which was measured using an Aloka 500 diagnostic real time ultrasound machine with a 17 cm 3.5 MHz linear array transducer. For slaughter, animals were first stunned by captive bolt and then exsanguinated. The animals were on average slaughtered at the age of 494 ± 3, 518 ± 4, and 457 ± 4 days for Angus, Charolais, and KC, respectively. The liver of each animal was collected immediately after slaughter and dissected at relatively the same location on the right lobe, and the fibrous capsule was removed from the sliced liver tissue samples. The sample tissues were further dissected into smaller portions that were bagged separately in plastic re-closable bags, labelled, flash frozen in liquid nitrogen, and transported on dry ice to the laboratory within 6 h, where they were stored at − 80 °C until total RNA extraction.

### Total RNA extraction

Total RNA extraction was performed on 20 of the liver tissue samples (10 with the highest and 10 with the lowest RFI phenotype values) from each breed as described in our recent studies^18,27^. Each of the selected liver tissue samples were pulverized into a fine powder using liquid nitrogen and a pre-chilled mortar and pestle on dry ice. Total RNA containing small RNAs was then extracted using the Qiagen RNeasy Plus Universal Mini Kit (Qiagen, Toronto, ON, USA) according to the manufacturer’s instructions. A NanoDrop 2000 Spectrophotometer (Thermo Scientific, Wilmington, DE, USA) was used to quantify the RNA. We obtained total RNA with an average concentration of 1851.8 ng/µl per sample, and with absorbance ratios (A260/280) ranging between 1.8 and 2.0. RNA integrity was confirmed using a TapeStation-Agilent instrument (Agilent Technologies Canada, Mississauga, ON, USA). RNA integrity number (RIN) values for all samples were higher than 8 which deemed them to be high quality and suitable for cDNA library preparation and downstream transcriptomic profiling.

### Construction of cDNA libraries and sequencing

In total 60 cDNA libraries were prepared and sequenced at the Clinical Genomics Centre (Toronto, ON, Canada) as described in our recent studies^18,27^. The libraries were prepared using the Illumina Truseq Small RNA Library Prep Kit (Illumina, San Diego, CA, USA) from 1 µg of total RNA. Initially, an RNA 3′ adapter was ligated to the 3′ ends of the RNAs in the total RNA samples using a T4 RNA Ligase 2 enzyme. Thereafter, an RNA 5′ adapter was added to the 5′ ends of the 3′ adaptor-ligated-RNAs using a T4 RNA Ligase. In this experiment, the RNA 3′ and RNA 5′ adapters were designed to specifically target miRNAs and other small RNAs resulting from similar biogenic processing. The 5′ and 3′ adapter ligated RNA was then reverse transcribed using the SuperScript II Reverse Transcriptase (Thermo Fisher Scientific, San Jose, CA, USA) and the RNA RT primer to generate single stranded cDNA. The cDNA was then PCR amplified with a universal RNA PCR primer, and a second RNA PCR primer containing a six-nucleotide indexing sequence to allow multiplexed sequencing of multiple samples on the same flow cell lane. The cDNA libraries were purified via gel electrophoresis using a 6% Novex TBE gel in Novex TBE running buffer which was run for 60 min at 145 V. Based on high resolution DNA ladder, the 160 bp and 145 bp cDNA bands were excised using a razor blade under UV light illumination for subsequent sequencing. Prior to sequencing, all the libraries were diluted to 2 nM. Thereafter equal volumes from each sample cDNA were used to construct four sequencing pools of 15 cDNA libraries each. The pooled cDNA libraries were sequenced on two flow cells using the Illumina Hiseq 2500 sequencing platform under Rapid run mode (Illumina, San Diego, CA, USA), with an expected read length of 50 bp [1 × 50 bp single read (SR)]. After sequencing, the raw sequence data were demultiplexed into individual FASTQ format files for each sample using the Illumina bcl2fastq-v2.17.1.14 conversion software (Illumina, San Diego, CA, USA). All phenotype and raw FASTQ format sequence data files have been deposited to the Gene Expression Omnibus (GEO) under the accession number GSE144432.

### Bioinformatic sequence data processing and miRNA expression profiling

Raw sequence reads were initially assessed for sequencing quality using FASTQC version 0.11.7^55^ as described in our recent studies^18,27^. The reads were evaluated for quality based on multiple parameters such as average read length, adaptor content, per sequence GC content, and per base sequence quality scores. Thereafter, the Illumina 3′ adaptor sequence (TGGAATTCTCGGGTGCCAAGG) was clipped from all the raw read sequences using cutadapt version 1.16^56^. In this study, reads of lengths shorter than 15 bp, or longer than 28 bp were removed as short or long reads, respectively. The retained reads were filtered for other bovine short RNA species including ribosomal RNAs (rRNAs), transfer RNAs (tRNAs), small nuclear RNAs (snRNAs), and small nucleolar RNAs (snoRNAs), downloaded from the Rfam database via RNAcentral a non-coding RNA database (https://rnacentral.org/search?q=expert_db:%22Rfam%22, accessed July 2018). The final processed sequence reads were re-evaluated for quality using FASTQC version 0.11.7^55^.

To profile both novel and known miRNA expression in the samples from the cleaned sequence data, the miRDeep2 package version 2.0.0.8 modules^33^ were used together with the UMD3.1 bovine genome from Ensembl version 93, the known bovine mature miRNA sequences and their precursor sequences from the miRBase database (release 22)^57^. The mapper module (mapper.pl) with default parameters was used to collapse reads of the sequences into clusters, and then bowtie-1.1.1 short sequence aligner^58^ was employed to align the collapsed reads to the indexed UMD3.1 reference genome. Using default parameters and input files including the reference genome, collapsed reads versus reference genome alignment, known bovine (and human) mature miRNAs and their precursor sequences (including the hairpin structures), and *Bos taurus* (bta) as the species of interest, all known bovine miRNAs were quantified by the miRDeep2 module (miRDeep2.pl), hence producing read counts for each individual sample.

Subsequently, miRDeep2 was used to predict possible novel miRNAs and their respective precursors based on their read alignment to the bovine reference genome^33^. Genomic regions stacked with aligned reads were excised as potential precursors and evaluated by the RNAfold tool within ViennaRNA-1.8.4^59^ for their potential to form stable secondary structures (hairpins), their ability to be partitioned into mature, loop and star strand, and their base pairing in the mature miRNA region. Subsequently, for each predicted miRNA, a mature miRNA consensus sequence, a precursor sequence, RNAfold P-value, the miRDeep2 score, and the probability that the predicted miRNA was a true positive were estimated and produced as outputs. Additionally, for each predicted novel miRNA, mirDeep2 outputs their hairpin structure of the precursor sequence and the read counts of each sample.

### Differential miRNA expression analysis

Initially, counts for each mature miRNA from more than one precursor were averaged. Thereafter, all miRNAs that had less than 10 total read counts across the studied samples within each breed were filtered out. Then miRNA expression variation patterns between 20 samples in each breed were visualized through a principle component analysis of the read counts from the mirDeep2 module using the DESeq2 Bioconductor package^60^ and the ggplot2 R^61^ package. We performed differential miRNA expression for all expressed known mature miRNAs and top 25 expressed novel miRNAs in each breed. Twelve samples including six samples with extreme high and six samples with extreme low-RFI phenotypes that showed consistent miRNA expression (as compared to all other samples in the same breed) were considered for differential miRNA expression using the egdeR Bioconductor package in R^62^. To reduce false positive rates of the analyses, miRNAs within samples from each of the breeds that had less than one count per million (CPM) in at least six samples (half of the analyzed samples) were filtered out from the analyses, as proposed by Anders et al.^63^. For the retained miRNAs, their counts were normalized using the TMM method^64^. To test for differential miRNA expression between high and low-RFI steer groups from each breed using the egdeR package in R^62^, the normalized counts were modeled using a generalized linear model under a negative binomial distribution with the high-RFI group as a reference. MicroRNAs were deemed differentially expressed (DE) at a P-value less than 0.05, and fold change (FC) greater than 1.5.

### Validation of differentially expressed miRNAs

Six differentially expressed miRNAs with relatively high expression levels per sample were selected for validation of the small RNAseq results. These included *bta-miR-2415-3p*, *bta-miR-133a,* and *bta-miR-2419-5p* for Charolais, and *bta-miR-424-5p*, *bta-miR-223,* and *bta-miR-155* for KC. *bta-miR-192* and *bta-miR-93* were selected as endogenous controls for Charolais whereas *bta-miR-2284* × and *bta-let-7b* were selected as reference miRNA genes for KC based on their expression abundance and stability across samples (average M values of 0.18 for KC and 0.22 for Charolais), which were determined using geNorm in the GenEx Software v.5.2.7.44 (MultiD Analyses AB, Gothenburg, Sweden). Relative expressions of the selected miRNAs were obtained through stem-loop RT-TaqMan qPCR^65^ from the same total RNA as was used for the small RNA sequencing. Reverse-transcription (RT) stem-loop primers and TaqMan qPCR assays (containing the probe and forward and reverse primers) were purchased from Thermo Fisher Scientific (https://www.thermofisher.com). RT primer IDs and TaqMan qPCR assay IDs for each validated internal control are provided in Table S7 of Supplementary File 1. Serial dilutions of pooled cDNA samples were used to determine amplification efficiencies using the equation E = − 1 + 10^(−1/slope)^, and the slope was calculated by plotting the linear curve of cycle threshold (C_T_) values against the log dilutions^66^. Primers had PCR efficiencies between 89 and 110%.

The reverse transcription reactions for each sample including no RNA template controls were performed using the TaqMan MicroRNA Reverse Transcription Kit. Each sample reaction contained 5 µl of total RNA (2 ng/µl), 1 µl of MultiScribe Reverse Transcriptase enzyme, 3 µl of stem-loop RT primer, 0.15 µl of dNTP mix, 1.5 µl of 10 × RT buffer, 0.19 µl of RNase inhibitor and 4.16 µl of nuclease free water. The 15 µl reactions were incubated in an Eppendorf 5331 Mastercycler Gradient v2.30.31 thermocycler (Marshall Scientific, Hampton, NH) for 30 min at 16 °C, 30 min at 42 °C and 5 min at 85 °C. Thereafter, real-time quantitative PCR (qPCR) was performed using the TaqMan Fast Advanced Master Mix Protocol. The 20 µl qPCR reaction contained 10 µl of TaqMan Fast Advanced Master Mix, 1 µl of TaqMan MicroRNA Assay, 1.33 µl of RT reaction product (cDNA), and 7.67 µl of nuclease free water. All qPCR reactions were performed in triplicate on a MicroAmp Fast Optical 96-Well Reaction Plate in the Applied Biosystems 7500 Fast Real-Time PCR System v2.0.1 (Applied Biosystems, Foster City, California, USA). The reactions were incubated for 2 min at 50 °C, for 20 s at 95 °C, and followed subsequently by 40 PCR cycles of 3 s at 95 °C for denaturation and 30 s at 60 °C for annealing and extension. Threshold cycle (C_T_) values from the Real-Time PCR thermocycler were then imported into GenEx Software v.5.2.7.44 (MultiD Analyses AB, Göteborg, Sweden). The C_T_ values were adjusted to account for inter-plate variation using the inter-plate calibrator sample included on the plates and to account for amplification efficiencies. The adjusted C_T_ values of the replicates were averaged, and then normalized to the endogenous controls of miRNA’s (reference genes) expression. Lastly Log_2_ relative quantities were calculated to the average (for all test samples) C_T_ value. The relative quantities were then analyzed for differential miRNA expression between the high and low-RFI steers using a two tailed t-test.

### miRNA target genes prediction and functional enrichment analyses

Target genes prediction was performed using three TargetScan version 7.0 Perl scripts^48^ downloaded from https://www.targetscan.org/cgi-bin/targetscan/data_download.vert72.cgi (Accessed July 2018) for the top expressed known miRNAs and top most expressed novel miRNAs that were commonly expressed across the three breeds. TargetScan predicts miRNA target genes based on a quantitative model that scores candidate target genes based on 14 features including 3′-UTR target-site abundance, predicted seed-pairing stability, identity of the nucleotide at position 1 of the small RNA (sRNA), identity of the nucleotide at the 8th position of the sRNA, identity of the nucleotide at the 8th position of the target site, local AU content near the target site, supplementary pairing at the miRNA 3′ end, predicted structural accessibility, minimum distance of the site from the stop codon or polyadenylation site, probability of target site conservation, open reading frame (ORF) length, 3′-UTR length, number of offset-6mer sites in the 3′ UTR, and the number of 8mer sites in the ORF^48^. Firstly, we predicted the conserved and non-conserved target sites using the targetscan_70.pl script by providing all the known gene transcripts UTR sequence alignments and the miRNA family information as inputs. The miRNA family information file included the miRNA family IDs, the seed sequence (from 2nd position nucleotide to 8th position nucleotide 5′ of miRNA sequences), and the NCBI IDs of 8 species (cow, sheep, domestic goat, horse, human, mouse, rat and pig). Thereafter, we used the targetscan_70_BL_bins.pl script and the targetscan_70_BL_PCT.pl script to calculate the branch length and the conservation probability of the conserved target sites. Finally, in combination with RNAplfold-2.4.11^59^, the targetscan_70_context_scores.pl script was used to calculate context++ scores for the miRNA target genes based mainly on the 14 attributes mentioned above. Subsequently, potential target genes for the most abundant miRNAs, and DE miRNAs of the 99th context++ score or > 50 context++ score percentile rank were considered for functional enrichment analysis using Ingenuity Pathway Analysis (IPA) web-based software (Redwood City, CA; https://www.qiagenbioinformatics.com/products/ingenuity-pathway-analysis/, IPA Spring 2019 release). In addition, Pearson correlation coefficients of most abundant miRNAs or DE miRNAs expression with mRNA expression of their predicted target genes that were expressed in the same animals of our previous study^18^ were calculated.

### Interaction network analyses between DE miRNA and DE genes

To explore potential interactions between DE miRNAs identified in this study and the DE genes between high and low-RFI animals that were reported in the same populations reported by Mukiibi et al.^18^, a context++ score greater than the 50 percentile rank for the DE miRNA target genes was used. For each breed, the DE genes identified as targets of the DE miRNAs at this threshold were retained for DE mRNA–DE miRNA (Target-miRNA) interaction network visualization using Cytoscape version 3.7.1^67^, and Pearson correlation coefficients of the DE miRNA expression with mRNA expression of the targeted DE genes were calculated. Furthermore, IPA was used to perform functional enrichment analyses for the targeted DE genes to identify major biological functions that are potentially differentially modulated by the identified DE miRNAs.

## Supplementary information

Supplementary Information 1.

Supplementary Information 2.

Supplementary Information 3.

Supplementary Information 4.

Supplementary Information 5.

Supplementary Information 6.

## References

[1] Ahola JK, Hill RA, Hill RA (2012). Input factors affecting profitability: A changing paradigm and a challenging time. Feed Efficiency in the Beef Industry.

[2] Hegarty R, Goopy JP, Herd R, McCorkell B (2007). Cattle selected for lower residual feed intake have reduced daily methane production. J. Anim. Sci..

[3] Nkrumah J (2006). Relationships of feedlot feed efficiency, performance, and feeding behavior with metabolic rate, methane production, and energy partitioning in beef cattle. J. Anim. Sci..

[4] Fang L (2017). Use of biological priors enhances understanding of genetic architecture and genomic prediction of complex traits within and between dairy cattle breeds. BMC Genom..

[5] Abo-Ismail MK (2014). Single nucleotide polymorphisms for feed efficiency and performance in crossbred beef cattle. BMC Genet..

[6] Barendse W (2007). A validated whole-genome association study of efficient food conversion in cattle. Genetics.

[7] de Oliveira PS (2014). Identification of genomic regions associated with feed efficiency in Nelore cattle. BMC Genet..

[8] Saatchi M (2014). QTLs associated with dry matter intake, metabolic mid-test weight, growth and feed efficiency have little overlap across 4 beef cattle studies. BMC Genom..

[9] Zhang F (2020). Genetic architecture of quantitative traits in beef cattle revealed by genome wide association studies of imputed whole genome sequence variants: I: Feed efficiency and component traits. BMC Genom..

[10] Alexandre PA (2015). Liver transcriptomic networks reveal main biological processes associated with feed efficiency in beef cattle. BMC Genom..

[11] Chen Y (2011). Global gene expression profiling reveals genes expressed differentially in cattle with high and low residual feed intake. Anim Genet..

[12] Higgins MG (2019). The effect of breed and diet type on the global transcriptome of hepatic tissue in beef cattle divergent for feed efficiency. BMC Genom..

[13] Kong RS, Liang G, Chen Y, Stothard P, Guan LL (2016). Transcriptome profling of the rumen epithelium of beef cattle difering in residual feed intake. BMC Genom..

[14] Paradis F (2015). Transcriptomic analysis by RNA sequencing reveals that hepatic interferon-induced genes may be associated with feed efficiency in beef heifers. J. Anim. Sci..

[15] Tizioto PC (2015). Global liver gene expression differences in Nelore steers with divergent residual feed intake phenotypes. BMC Genom..

[16] Tizioto PC (2016). Gene expression differences in Longissimus muscle of Nelore steers genetically divergent for residual feed intake. Sci. Rep..

[17] Weber KL (2016). Identification of gene networks for residual feed intake in Angus cattle using genomic prediction and RNA-seq. PLoS ONE.

[18] Mukiibi R (2018). Transcriptome analyses reveal reduced hepatic lipid synthesis and accumulation in more feed efficient beef cattle. Sci. Rep..

[19] Gregory RI, Shiekhattar R (2005). MicroRNA biogenesis and cancer. Cancer Res..

[20] Moutinho C, Esteller M (2017). MicroRNAs and epigenetics. Adv. Cancer Res..

[21] Bartel DP (2018). Metazoan micrornas. Cell.

[22] O'Brien J, Hayder H, Zayed Y, Peng C (2018). Overview of microRNA biogenesis, mechanisms of actions, and circulation. Front. Endocrinol..

[23] Creighton CJ, Reid JG, Gunaratne P (2009). Expression profiling of microRNAs by deep sequencing. Brief Bioinform..

[24] Friedman RC, Farh KKH, Burge CB, Bartel DP (2009). Most mammalian mRNAs are conserved targets of microRNAs. Genome Res..

[25] Murakami Y, Kawada N (2017). MicroRNAs in hepatic pathophysiology. Hepatol. Res..

[26] Chen Y, Verfaillie CM (2014). Micro RNAs: The fine modulators of liver development and function. Liver Int..

[27] Mukiibi R (2019). Liver transcriptome profiling of beef steers with divergent growth rate, feed intake, or metabolic body weight phenotypes. J. Anim. Sci..

[28] Al-Husseini W (2016). Characterization and profiling of liver microRNAs by RNA-sequencing in cattle divergently selected for residual feed intake. Asian Aust. J. Anim. Sci..

[29] De Oliveira PS (2018). An integrative transcriptome analysis indicates regulatory mRNA-miRNA networks for residual feed intake in Nelore cattle. Sci. Rep..

[30] Carvalho EB (2019). Differentially expressed mRNAs, proteins and miRNAs associated to energy metabolism in skeletal muscle of beef cattle identified for low and high residual feed intake. BMC Genom..

[31] Motameny S, Wolters S, Nürnberg P, Schumacher B (2010). Next generation sequencing of miRNAs—strategies, resources and methods. Genes..

[32] Pritchard CC, Cheng HH, Tewari M (2012). MicroRNA profiling: Approaches and considerations. Nat. Rev. Genet..

[33] Friedländer MR, Mackowiak SD, Li N, Chen W, Rajewsky N (2012). miRDeep2 accurately identifies known and hundreds of novel microRNA genes in seven animal clades. Nucleic Acids Res..

[34] Sun H-Z, Chen Y, Guan LL (2019). MicroRNA expression profiles across blood and different tissues in cattle. Sci. Data..

[35] Morimoto A (2017). An HNF4α–microRNA-194/192 signaling axis maintains hepatic cell function. J. Biol. Chem..

[36] Zhao Q (2016). Expression profiling and functional characterization of miR-192 throughout sheep skeletal muscle development. Sci. Rep..

[37] Jordan SD (2011). Obesity-induced overexpression of miRNA-143 inhibits insulin-stimulated AKT activation and impairs glucose metabolism. Nat. Cell Biol..

[38] Zhou J (2012). Down-regulation of microRNA-26a promotes mouse hepatocyte proliferation during liver regeneration. PLoS ONE.

[39] Gailhouste L (2013). miR-148a plays a pivotal role in the liver by promoting the hepatospecific phenotype and suppressing the invasiveness of transformed cells. Hepatology.

[40] Takagi S, Nakajima M, Mohri T, Yokoi T (2008). Post-transcriptional regulation of human pregnane X receptor by micro-RNA affects the expression of cytochrome P450 3A4. J. Biol. Chem..

[41] Bates DJ (2010). MicroRNA regulation in Ames dwarf mouse liver may contribute to delayed aging. Aging Cell.

[42] Tao Y, Wang M, Chen E, Tang H (2017). Liver regeneration: Analysis of the main relevant signaling molecules. Mediat. Inflamm..

[43] Cordero-Espinoza L, Huch M (2018). The balancing act of the liver: Tissue regeneration versus fibrosis. J. Clin. Investig..

[44] Chen X (2015). MicroRNAs in liver regeneration. Cell Physiol. Biochem..

[45] Yi P-S, Zhang M, Xu M-Q (2016). Role of microRNA in liver regeneration. Hepatobiliary Pancreat. Dis. Int..

[46] Häussinger D, Greger R, Windhorst U (1996). Physiological functions of the liver. Comprehensive Human Physiology.

[47] Parker GA, Picut CA (2005). Liver immunobiology. Toxicol. Pathol..

[48] Agarwal V, Bell GW, Nam J-W, Bartel DP (2015). Predicting effective microRNA target sites in mammalian mRNAs. elife.

[49] Hashimoto Y, Akiyama Y, Yuasa Y (2013). Multiple-to-multiple relationships between microRNAs and target genes in gastric cancer. PLoS ONE.

[50] Stroynowska-Czerwinska A, Fiszer A, Krzyzosiak WJ (2014). The panorama of miRNA-mediated mechanisms in mammalian cells. Cell Mol. Life Sci..

[51] CCAC. CCAC guidelines on: the care and use of farm animals in research, teaching and testing. (ed. Care, C.C.o.A.) (Canadian Council on Animal Care, Ottawa, Ontario, 2009). https://www.ccac.ca/Documents/Standards/Guidelines/Farm_Animals.pdf. Accessed 1 Feb 2020.

[52] Goonewardene L (2003). Effect of udder type and calving assistance on weaning traits of beef and dairy× beef calves. Livest. Prod. Sci..

[53] Mao F (2013). Phenotypic and genetic relationships of feed efficiency with growth performance, ultrasound, and carcass merit traits in Angus and Charolais steers. J. Anim. Sci..

[54] Koch RM, Swiger LA, Chambers D, Gregory KE (1963). Efficiency of feed use in beef cattle. J. Anim. Sci..

[55] Andrews, S. FastQC: A quality control tool for high throughput sequence data. https://www.bioinformatics.babraham.ac.uk/projects/fastqc/ (2010). Accessed 18 June 2019.

[56] Martin M (2011). Cutadapt removes adapter sequences from high-throughput sequencing reads. EMBnet J..

[57] Griffiths-Jones S, Saini HK, van Dongen S, Enright AJ (2007). miRBase: Tools for microRNA genomics. Nucleic Acids Res..

[58] Langmead B, Trapnell C, Pop M, Salzberg SL (2009). Ultrafast and memory-efficient alignment of short DNA sequences to the human genome. Genome Biol..

[59] Lorenz R (2011). ViennaRNA Package 2.0. Algorithms Mol. Biol..

[60] Love MI, Huber W, Anders S (2014). Moderated estimation of fold change and dispersion for RNA-seq data with DESeq2. Genome Biol..

[61] Wickham, H., Chang, W. & Wickham, M. H. Package ‘ggplot2’. Create elegant data visualisations using the grammar of graphics. Version 3.2.1. pp. 1–227. https://cloud.r-project.org/web/packages/ggplot2/ggplot2.pdf. Accessed 18 June 2019.

[62] Robinson MD, McCarthy DJ, Smyth GK (2010). edgeR: A Bioconductor package for differential expression analysis of digital gene expression data. Bioinformatics.

[63] Anders S (2013). Count-based differential expression analysis of RNA sequencing data using R and Bioconductor. Nat. Protoc..

[64] Robinson MD, Oshlack A (2010). A scaling normalization method for differential expression analysis of RNA-seq data. Genome Biol..

[65] Chen C (2005). Real-time quantification of microRNAs by stem-loop RT–PCR. Nucleic Acids Res..

[66] Pfaffl MW (2001). A new mathematical model for relative quantification in real-time RT–PCR. Nucleic Acids Res..

[67] Shannon P (2003). Cytoscape: A software environment for integrated models of biomolecular interaction networks. Genome Res..

