# Supplementary material for: Bovine hepatic miRNAome profiling and differential miRNA expression analyses between beef steers with divergent feed efficiency phenotypes
Source: Sci Rep. 2020 Nov 9;10:19309. doi: 10.1038/s41598-020-73885-5 (PMC7653039; doi:10.1038/s41598-020-73885-5)
Supplement: Supplementary file 1 — Supplementary Information 1. [file 41598_2020_73885_MOESM1_ESM.docx]

**Bovine hepatic miRNAome profiling and differential miRNA expression analyses between beef steers with divergent feed efficiency phenotypes**

Robert Mukiibi^1^, Dayle Johnston^2^, Michael Vinsky^3^, Carolyn Fitzsimmons^1, 3^, Paul Stothard^1^, Sinéad M. Waters^2,*^ & Changxi Li^1, 3,*^

^1^Department of Agricultural, Food and Nutritional Science, University of Alberta, Edmonton, Alberta, Canada, T6G 2P5. ^2^Animal and Bioscience Research Department, Teagasc, Grange, Dunsany, County Meath, Ireland. ^3^Lacombe Research and Development Centre, Lacombe, Agriculture and Agri-Food Canada, Alberta, Canada, T4L 1W1

Correspondence and requests for materials should be addressed to Changxi Li (email: [changxi.li@canada.ca](mailto:changxi.li@canada.ca) ) or Sinead Waters ([sinead.waters@teagasc.ie](mailto:sinead.waters@teagasc.ie))

|  | **Angus** | **Charolais** | **Kinsella Composite (KC)** |
| --- | --- | --- | --- |
| **Before quality control** |  |  |  |
| Average no. of reads | 9,450,928 (1,551,281) | 9,620,729 (1,034,563) | 11,230,561 (1,128,159) |
| Read length/bp | 51 | 51 | 51 |
| Average quality score | 37.30 (0.87) | 37.00 (0.91) | 37.64 (0.87) |
| **Quality control processing** |  |  |  |
| Long reads (Length > 28bp (%)) | 43.06 (10.53) | 33.83 (11.19) | 33.44 (10.17) |
| Short reads (Length < 15bp (%)) | 7.92 (3.48) | 8.74 (2.31) | 7.90 (2.20) |
| Read aligned to rRNA (%) | 0.24 (0.12) | 0.25 (0.06) | 0.24 (0.07) |
| Read aligned to tRNA (%) | 0.14 (0.06) | 0.15 (0.01) | 0.14 (0.03) |
| Read aligned to snRNA (%) | 0.03 (0.01) | 0.03 (0.01) | 0.03 (0.01) |
| Read aligned to snoRNA (%) | 0.03 (0.01) | 0.03(0.01) | 0.03 (0.01) |
| Retained Reads (%) | 48.58 (3.24) | 56.98(10.22) | 58.22 (10.02) |
| **Post quality control process quality** |  |  |  |
| Average number of reads | 4,553,319 (1,122,740) | 5,497,788 (1,192,534) | 6,483,795 (993,382) |
| Average read length (bp) | 22(2) | 21 (2) | 21 (2) |
| Mapping/alignment rate (%) | 74.77 (3.24) | 72.47 (1.20) | 77.09 (1.34) |

**Table S1:** Sequence data quality assessment, quality control processing and alignment summaries, standard deviations are presented in parentheses or ().

|  | **Angus** |  |  | **Charolais** |  |  | **Kinsella Composite (KC)** | | |
| --- | --- | --- | --- | --- | --- | --- | --- | --- | --- |
| **Trait** | **H_RFI±SE** | **L_RFI±SE** | **P-value** | **H_RFI±SE** | **L_RFI±SE** | **P-value** | **H_RFI±SE** | **L_RFI±SE** | **P-value** |
| RFI/kg/day | 1.26±0.11 | -0.84±0.07 | 1.13E-08 | 1.15±0.16 | -0.98±0.09 | 4.19E-07 | 1.52±0.12 | -1.29±0.11 | 1.18E-08 |
| DMI/kg/day | 12.97±0.35 | 11.46±0.51 | 0.04 | 12.23±0.19 | 10.23±0.12 | 4.18E-06 | 12.74±0.36 | 9.21±0.36 | 3.95E-05 |
| ADG/kg/day | 1.6±0.07 | 1.88±0.11 | 0.06 | 1.68±0.09 | 1.62±0.05 | 0.62 | 1.63±0.07 | 1.48±0.1 | 0.26 |
| MWT/kg | 115.09±2.56 | 115.58±5.41 | 0.94 | 118.39±2.01 | 121.17±1.42 | 0.29 | 104.67±2.77 | 99.7±2.7 | 0.23 |
| FUREA/cm^2^ | 79.72±3.06 | 84.41±1.56 | 0.20 | 88.86±2.41 | 99±2.7 | 0.02 | 74.22±1.52 | 71.02±2.77 | 0.34 |
| FUFAT/kg | 9.89±0.62 | 9.23±0.68 | 0.49 | 5.67±0.63 | 6.69±0.91 | 0.38 | 8.98±0.45 | 8.75±0.55 | 0.75 |
| HCW/lb | 754.78±22.47 | 763.23±44.26 | 0.87 | 828.17±10.5 | 874.17±19.67 | 0.07 | 697.33±24.54 | 656.67±21.52 | 0.24 |
| AFAT/mm | 12.33±1.33 | 10.67±1.09 | 0.36 | 6.67±0.49 | 7.67±1.28 | 0.20 | 10±0.52 | 11.67±1.17 | 0.22 |
| CREA/cm^2^ | 72±3.79 | 75.83±2.34 | 0.41 | 89.83±2.96 | 97.83±4.95 | 0.20 | 76.33±2.23 | 69.67±2.54 | 0.08 |
| LMY/% | 54.59±1.56 | 56.43±1.18 | 0.37 | 61.28±0.77 | 61.63±1.32 | 0.82 | 57.81±0.56 | 55.79±0.88 | 0.08 |
| Marbling score | 448.33±24.95 | 393.33±23.47 | 0.14 | 393.33±17.44 | 353.33±16.46 | 0.13 | 378.33±20.56 | 378.33±20.56 | 1 |
| Slaughter age/day | 489.33±5.29 | 500.33±4.38 | 0.14 | 512.5±9.11 | 519.67±4.14 | 0.49 | 445.17±3.44 | 464±7.08 | 0.04 |

**Table S2:** “*” indicates significant difference (P-value < 0.0042, Bonferroni Correction for 12 multiple testing analyses at P<0.05). DMI = daily dry matter intake , RFI = residual feed intake, ADG = average daily gain, MWT = metabolic body weight, FUREA = final ultrasound ribeye area at the end of feedlot test; FUFAT = final ultrasound backfat at the end of feedlot test; HCW = hot carcass weight; AFAT = carcass average backfat; REA = carcass ribeye area; LMY = lean meat yield; Marbling score (100–399 = trace marbling or less, 400–499 = slight marbling, 500–799 = small to moderate marbling, and 800–1199 = slightly abundant or more marbling). L_RFI ± SE= trait mean values for the low RFI group± standard error (SE); H_RFI ± SE= trait mean values for the high RFI steer group ± standard error (SE).

|  | **Biological function** | **No. of DE targets** | **Targets involved in the biological function** |
| --- | --- | --- | --- |
| **Angus** | Lipid metabolism | 18 | *ACSS2, ADIPOR2, CCDC80, CPT1B, DLK1, EDNRA, ELOVL5, FKBP5, G0S2, GATM, HP, LPIN1, MARCO, PLA2G2D, SCD, SLC22A2, UGT2B7, ZBTB16* |
|  | Molecular transport | 16 | *ADIPOR2, CCDC80, CPT1B, DLK1, EDNRA, ELOVL5, G0S2, GATM, HP, LPIN1, MARCO, PLA2G2D, SCD, SLC22A2, TP53INP1, ZBTB16* |
|  | Small molecule biochemistry | 21 | *ACSS2, ADIPOR2, CCDC80, CPT1B, DLK1, EDNRA, ELOVL5, FKBP5, G0S2, GATM, HP, LPIN1, LURAP1L, MARCO, PLA2G2D, SCD, SLC22A2, SLCO4A1, TP53INP1, UGT2B7, ZBTB16* |
|  | Energy production | 7 | *ACSS2, ADIPOR2, CCDC80, CPT1B, G0S2, LPIN1, SCD* |
|  | Carbohydrate metabolism | 9 | *ADIPOR2, CCDC80, ELOVL5, GATM, GNAZ, LPIN1, PLA2G2D, SCD, TP53INP1* |
| **Charolais** | Lipid metabolism | 12 | *ABCC4, CES1, CYP2C19, DLK1, LPIN1, NR0B2, PDK4, SCD, SLC4A4, SPNS2, THEM4, TNC* |
|  | Molecular transport | 16 | *ABCC4, ANXA2, CES1, CXCL2, DLK1, LPIN1, NR0B2, PDK4, SCD, SIRPA, SLC13A2, SLC4A4, SLC7A5, SPNS2, TNC, TP53INP1* |
|  | Small molecule biochemistry | 19 | *ABCC4, ANXA2, CES1, CYP2C19, DLK1, GPX3, LPIN1, MIOX, NR0B2, PDK4, SCD, SLC13A2, SLC4A4, SLC7A5, SPNS2, THEM4, TNC, TP53INP1* |
|  | Cellular movement | 13 | *ABCC4, ANXA2, CES1, CXCL2, GNAZ, GPNMB, PDK4, SERPINA3, SIRPA, SLC7A5, SPNS2, TNC, TP53INP1* |
|  | Cell-to-cell signalling and interaction | 16 | *ABCC4, ANXA2, CES1, CXCL2, CYP2C19, DLK1, GNAZ, GPNMB, GPX3, HLA-DQB1, PDK4, RND1, SIRPA, SLC4A4, SLC7A5, TNC* |
| **KC** | Cell death and survival | 48 | *ACACA, APMAP, ARG1, ATP2A2, BAG3, BTG2, CCND1, CXCL10, CYCS, DDIT4, DUSP1, EDNRA, ERBB2, ERBB3, FGF21, FKBP5, GATA4, GCH1, GCLC, GHR, GLS2, HEYL, HMGCR, IGF1, INSIG1, IRAK3, KYAT1, LRIG1, MANF, MFSD2A, MKNK1, MOB3B, NMNAT2, NPC1, NR0B2, OAS1, PER1, PNP, PPARGC1A, RHOJ, RRS1, SCD, SERPINA3, TOP1, TP53INP1, TRIB2, USP2, ZBTB16* |
|  | Amino acid metabolism | 15 | *ACMSD, ARG1, GCH1, GCLC, GLS2, HAL, IGF1, KYAT1, OAT, SDS, SLC16A10, SLC22A7, SLC25A15, SLC7A2, TAT* |
|  | Small molecule biochemistry | 57 | *ABCG8, ACACA, ACMSD, AK4, ARG1, ATP2A2, BAG3, CXCL10, CYCS, CYP1A1, CYP2B6, DUSP1, EDNRA, ELOVL2, ERBB2, ERBB3, FGF21, FOXA3, GATA4, GCH1, GCLC, GHR, GLS2, GSTM4, HAL, HMGCR, IGF1, INSIG1, KYAT1, LPIN1, MFSD2A, MKNK1, NMNAT2, NPC1, NR0B2, OAS1, OAT, OGDH, P2RY2, PER1, PNP, PPARGC1A, RHOJ, SCD, SDS, SLC16A10, SLC22A7, SLC25A15, SLC7A2, SLCO4A1, STS, TAT, TP53INP1, TPH1, USP2, ZBTB16* |
|  | Lipid metabolism | 32 | *ABCG8, ACACA, ATP2A2, BAG3, CXCL10, CYCS, CYP1A1, CYP2B6, DUSP1, EDNRA, ELOVL2, ERBB2, FGF21, GATA4, GHR, GSTM4, HMGCR, IGF1, INSIG1, LPIN1, MFSD2A, MKNK1, NPC1, NR0B2, OGDH, P2RY2, PER1, PPARGC1A, RBP5, SCD, STS, ZBTB16* |
|  | Vitamin and mineral metabolism | 16 | *ABCG8, ACACA, ACMSD, CXCL10, CYP1A1, CYP2B6, GCLC, HMGCR, IGF1, INSIG1, NPC1, NR0B2, PPARGC1A, RBP5, SCD, STS* |

**Table S3**. Top five molecular and cellular (biological) functions enriched by DE target genes for Angus, Charolais, and Kinsella Composite (KC) populations.

| **miRNA** | **Fold-Change** | **logFC** | **P-value** | **Breed** |
| --- | --- | --- | --- | --- |
| *bta-miR-2415-3p* | 2.135 | 1.095 | 0.144 | Charolais |
| *bta-miR-133a* | 1.555 | 0.637 | 0.003 | Charolais |
| *bta-miR-2419-5p* | 1.521 | 0.605 | 0.273 | Charolais |
| *bta-miR-424-5p* | 1.358 | 0.441 | 0.086 | Kinsella Composite **(**KC) |
| *bta-miR-223* | 1.350 | 0.433 | 0.054 | Kinsella Composite **(**KC) |
| *bta-miR-155* | 1.852 | 0.889 | 0.314 | Kinsella Composite (KC) |

**Table S4:** qPCR validation results showing the log_2_(Fold-Change) or logFC, and the P-values for the t-tests between relative expression differences between high and low-RFI steers**.** Fold changes and log_2_(Fold-Change) show miRNA expression in low-RFI steers liver tissue relative to high-RFI.

|  | **miRNA** | **RNAseq-qPCR profile correlations** | **P-value** |
| --- | --- | --- | --- |
| **Charolais** | *bta-miR-2415-3p* | 0.93 | 1.00E-05 |
|  | *bta-miR-2419-5p* | 0.91 | 3.00E-05 |
|  | *bta-miR-133a* | 0.88 | 1.60E-04 |
|  |  |  |  |
| **KC** | *bta-miR-155* | 0.99 | 6.00E-10 |
|  | *bta-miR-223* | 0.94 | 5.87E-06 |
|  | *bta-miR-424-5p* | 0.73 | 6.79E-03 |

**Table S5**: RNAseq-qPCR profile correlations for the six validation miRNAs in Charolais and KC samples.

| **Nutrient component** | **Composition/content (Mean±SE**) |
| --- | --- |
| Moisture | 4.45±0.13 |
| Dry Matter - DM | 95.55±0.13 |
| Crude Protein - CP | 15.25±0.41 |
| Adjusted Protein | 15.25±0.41 |
| Soluble Protein | 5.43±0.63 |
| Acid Detergent Fiber -ADF | 18.70±2.27 |
| Neutral detergent fiber- NDF | 34.10±2.7 |
| Ash (%DM) | 8.94±0.95 |
| Calcium (%DM) | 1.18±0.16 |
| Phosphorus (%DM) | 0.46±0.02 |
| Magnesium (%DM) | 0.25±0.01 |
| Potassium (%DM) | 0.96±0.11 |
| Sodium (%DM) | 0.30±0.07 |
| Iron (PPM) | 527.75±95.42 |
| Manganese (PPM) | 132.50±27.44 |
| Zinc (PPM) | 200.75±27.64 |
| Copper (PPM) | 32.00±6.18 |
| Total digestible nutrient- TDN (%DM) | 68.60±1.84 |
| Non-Fiber Carbohydrates (%DM) | 41.75±3.66 |

**Table S6:** Average nutrient compositions of the finishing diet. PPM = Parts per million and SE = standard error.

| **No.** | **miRNA (miRBase ID)** | **miRBase accession Number** | **RNAseq DE or Ref** | **Thermo Fisher Scientific Assay ID** | **Thermo Fisher Scientific Catalog Number** | **Context Sequence** |
| --- | --- | --- | --- | --- | --- | --- |
| 1 | *bta-miR-2415-3p* | MIMAT0011979 | RNAseq DE | 241869_mat | 4440886 | CCAGGCCUGCUGGACCGACGC |
| 2 | *bta-miR-133a* | MIMAT0009225 | RNAseq DE | 002246 | 4427975 | UUUGGUCCCCUUCAACCAGCUG |
| 3 | *bta-miR-2419-5p* | MIMAT0011985 | RNAseq DE | 244440_mat | 4440886 | AUCGCAUCAACACUCGUCUGUU |
| 4 | *bta-miR-424-5p* | MIMAT0013593 | RNAseq DE | 242183_mat | 4440886 | CAGCAGCAAUUCAUGUUUUGA |
| 5 | *bta-miR-223* | MIMAT0009270 | RNAseq DE | 002295 | 4427975 | UGUCAGUUUGUCAAAUACCCCA |
| 6 | *bta-miR-155* | MIMAT0009241 | RNAseq DE | 002623 | 4427975 | UUAAUGCUAAUCGUGAUAGGGGU |
| 7 | *bta-miR-192* | MIMAT0003820 | Ref | 006776_mat | 4427975 | CUGACCUAUGAAUUGACAGCCAG |
| 8 | *bta-miR-93* | MIMAT0003837 | Ref | 007615_mat | 4440886 | CAAAGUGCUGUUCGUGCAGGUA |
| 9 | *bta-miR-228x* | MIMAT0017395 | Ref | 464990_mat | 4440886 | UGAAAAGUUCGUUCGGGUUUU |
| 10 | *bta-let-7b* | MIMAT0004331 | Ref | 002619 | 4427975 | UGAGGUAGUAGGUUGUGUGGUU |

**Table S7:** All miRNAs used in the qPCR validation with their Thermo Fisher Scientific specifications, RNAseq DE = differentially expressed miRNA from RNAseq analyses, Ref = reference miRNA.


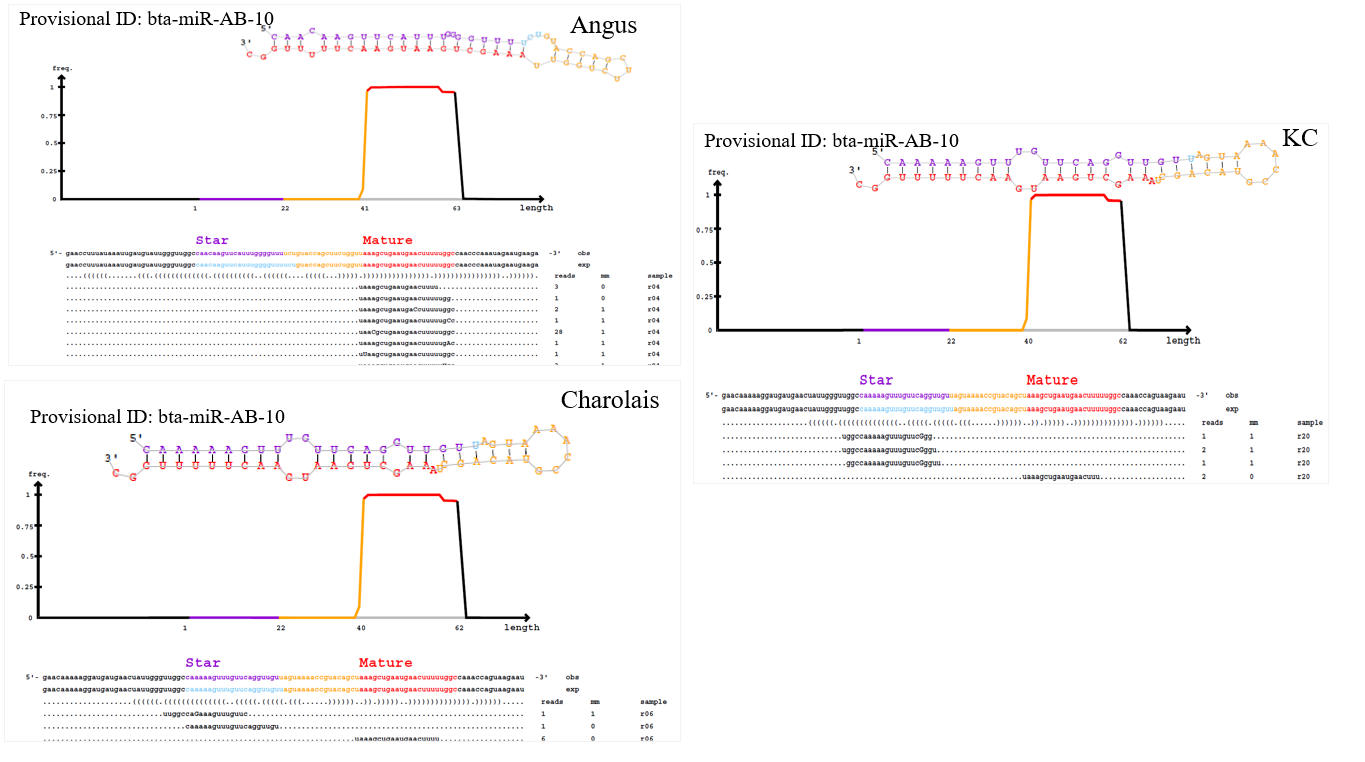


**Figure S1:** Precursor hairpin structure and alignment summary of the most expressed novel miRNA (*bta-miR-AB-10*) across liver tissue of the three studied populations (Angus, Charolais, and KC steers).


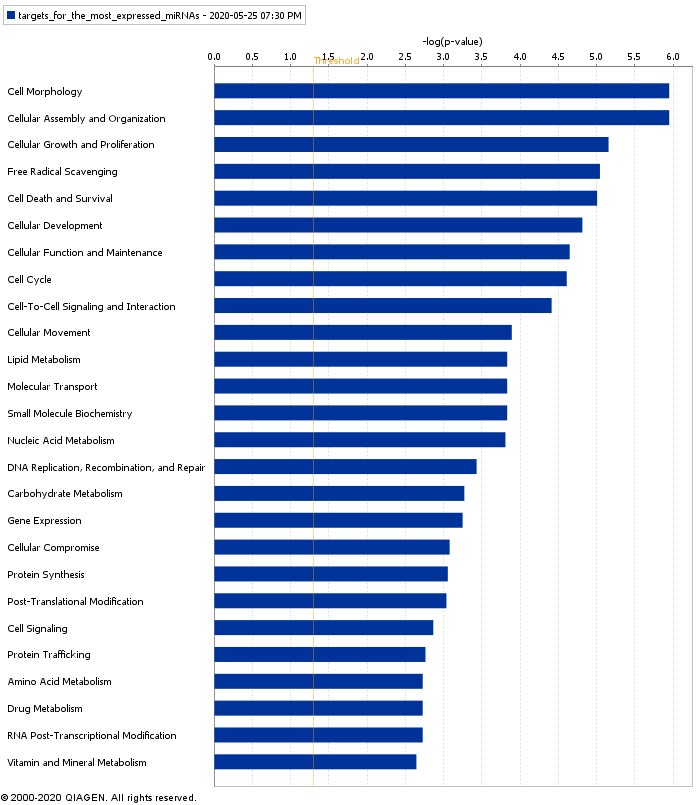


**Figure S2:** All enriched cellular and molecular functions enriched by target genes for the 18 most expressed miRNAs (16 known and 2 novel miRNAs) across the liver tissues of steers from the three populations. The image was generated from Ingenuity Pathway Analysis (IPA).


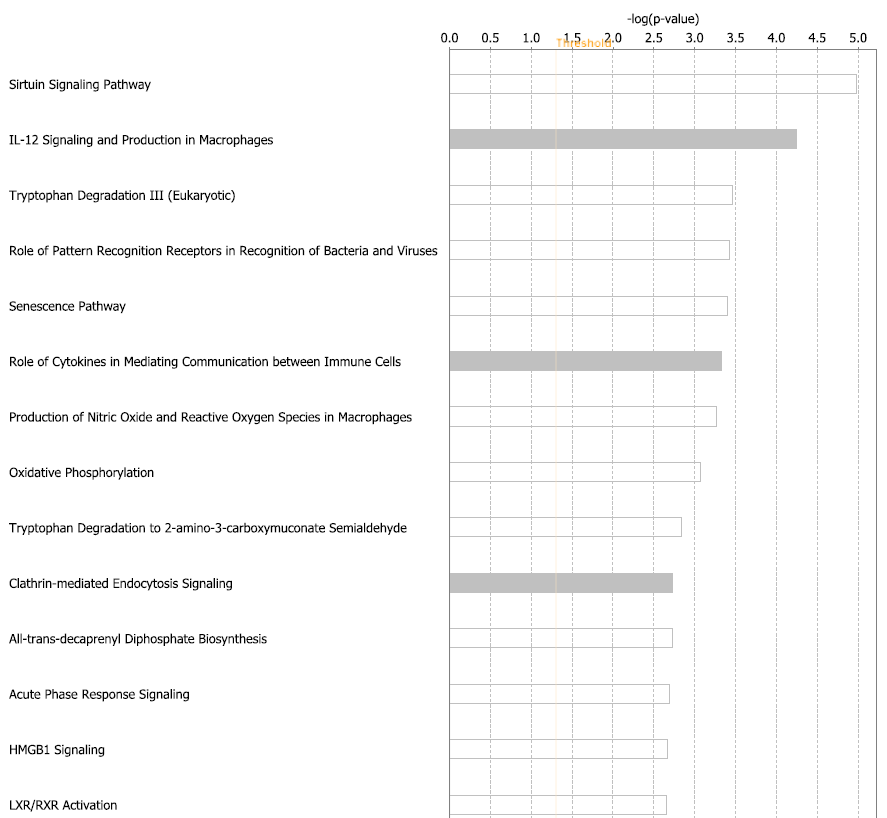


**Figure S3:** Fourteen topmost significantly enriched canonical pathways enriched by target genes of the 18 most expressed miRNAs (16 known and 2 novel miRNAs) across the liver tissues of steers from the three populations. The image was generated from Ingenuity Pathway Analysis (IPA).


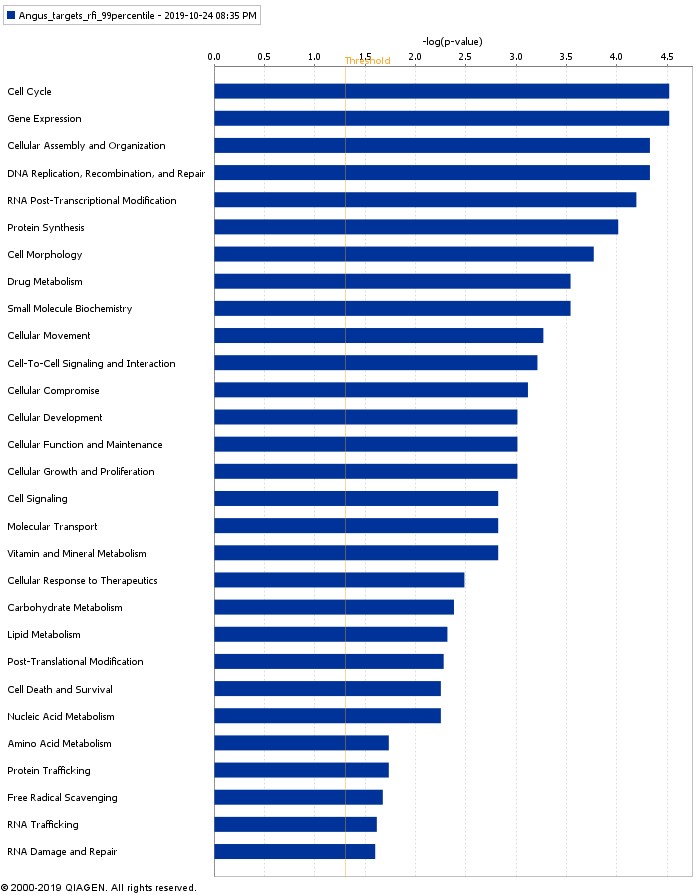


**Figure S4**: All cellular and molecular functions enriched by DE miRNAs’ targets at a TargetScan context++ score percentile threshold of 99 and above in Angus steers, generated from Ingenuity Pathway Analysis (IPA).


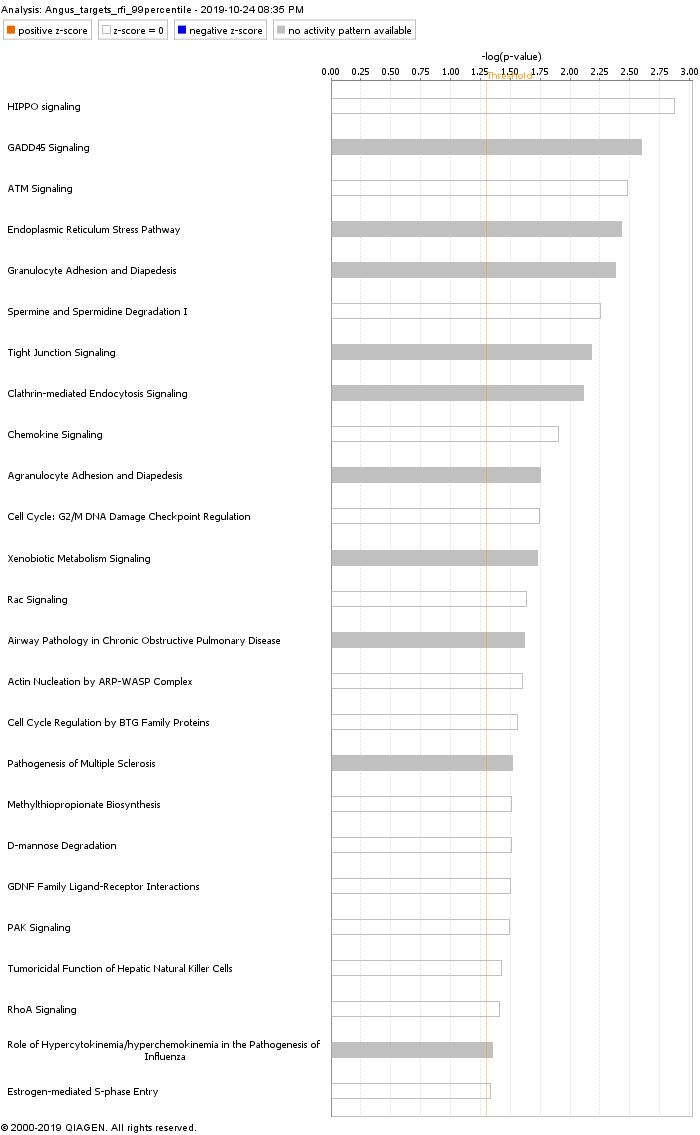


**Figure S5**: All canonical pathways enriched by DE miRNAs’ targets at a TargetScan context++ score percentile threshold of 99 and above in Angus steers, generated from Ingenuity Pathway Analysis (IPA).


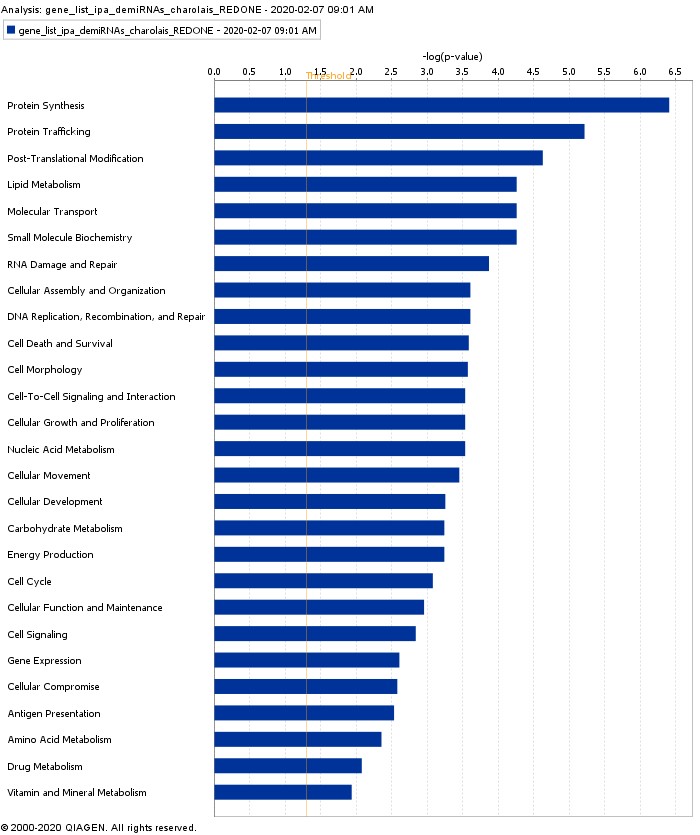


**Figure S6**: All cellular and molecular functions enriched by DE miRNAs’ targets at a TargetScan context++ score percentile threshold of 99 and above in Charolais steers, generated from Ingenuity Pathway Analysis (IPA).


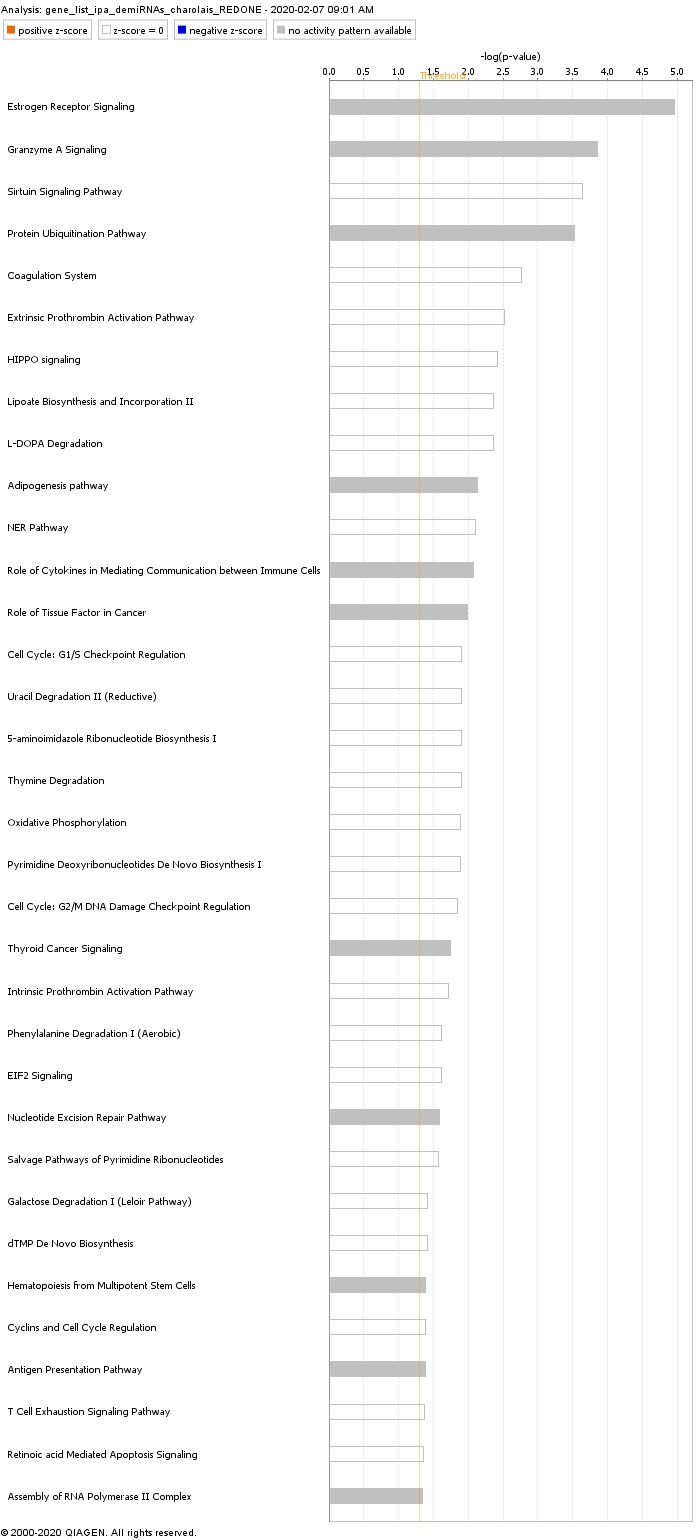
**Figure S7**: All canonical pathways enriched by DE miRNAs’ targets at a TargetScan context++ score percentile threshold of 99 and above in Charolais steers, generated from Ingenuity Pathway Analysis (IPA).


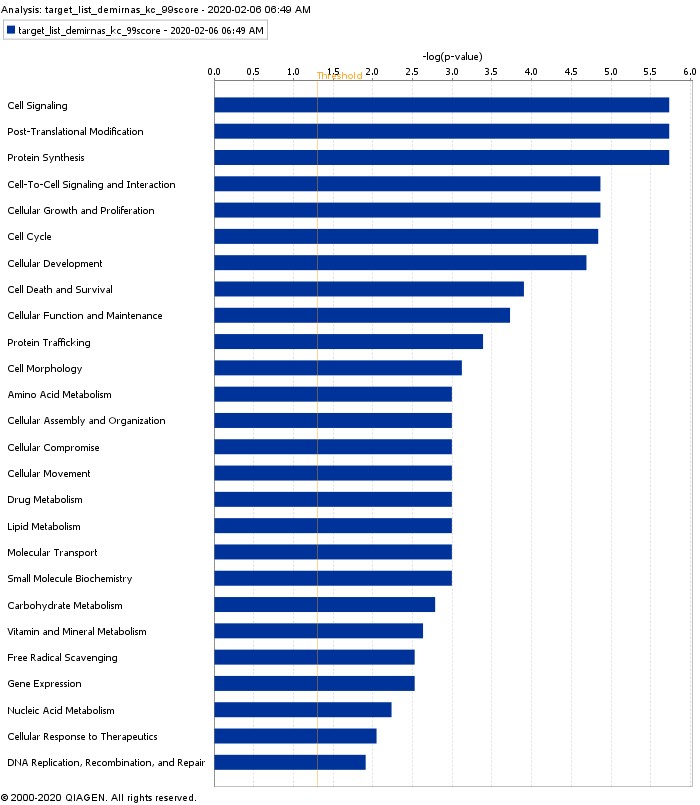


**Figure S8**: All cellular and molecular functions enriched by DE miRNAs’ targets at a TargetScan context++ score percentile threshold of 99 and above in KC steers, generated from Ingenuity Pathway Analysis (IPA).


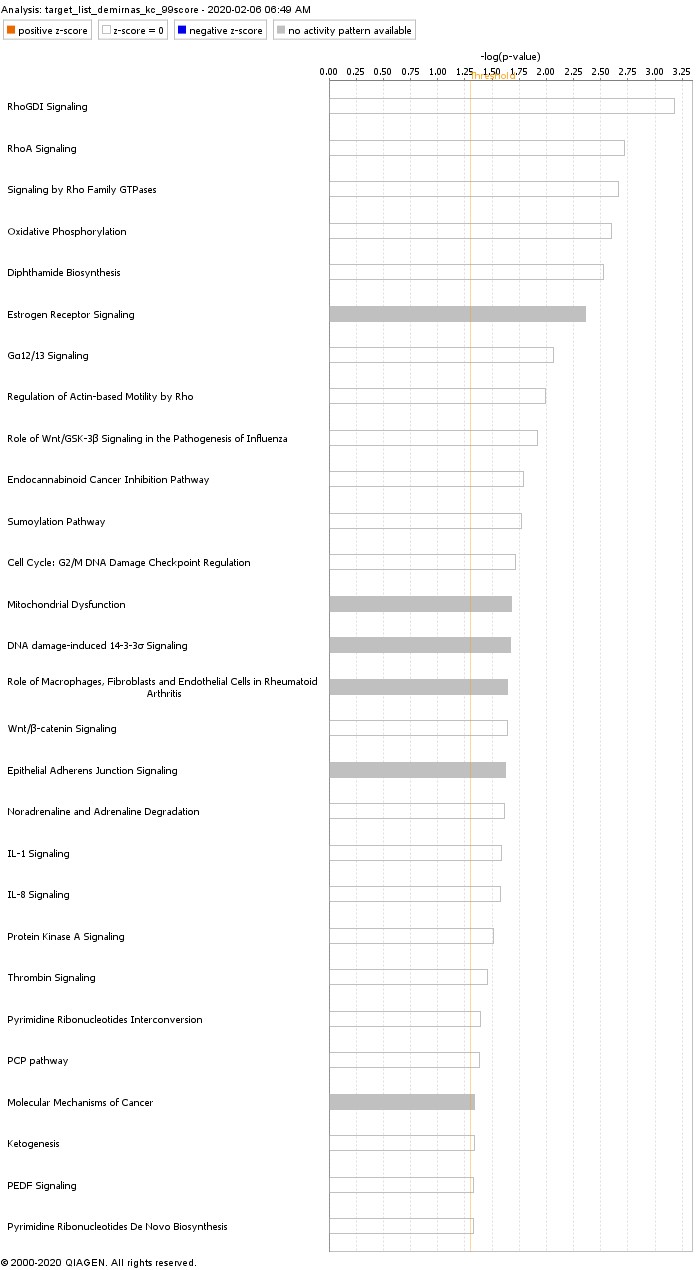


**Figure S9**: All canonical pathways enriched by DE miRNAs’ targets at a TargetScan context++ score percentile threshold of 99 and above in KC steers, generated from Ingenuity Pathway Analysis (IPA).


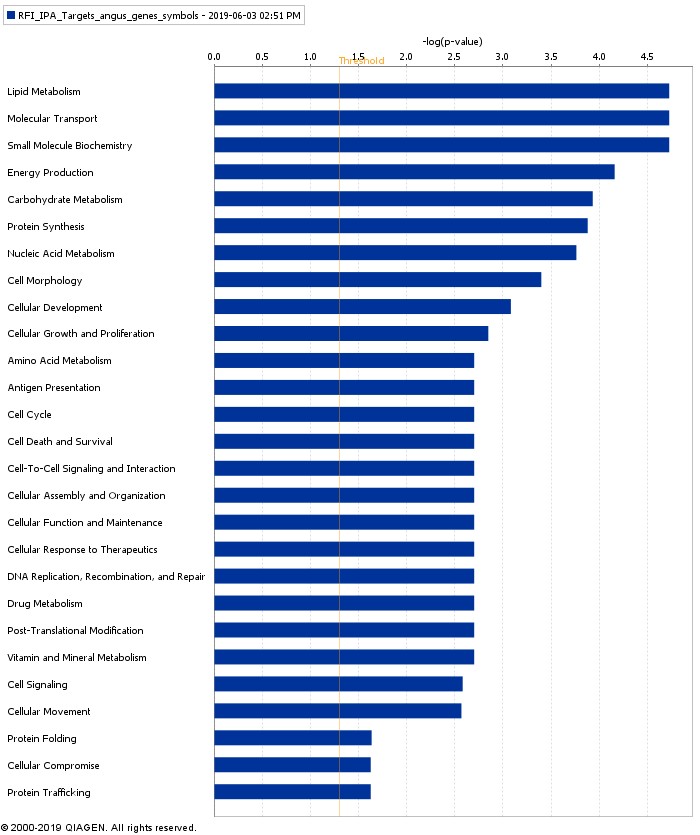


**Figure S10**: All cellular and molecular functions enriched by DE miRNAs’ DE targets (as previously reported by Mukiibi et al. (2018)) at a TargetScan context++ score percentile threshold of greater than 50 in Angus steers, generated from Ingenuity Pathway Analysis (IPA).


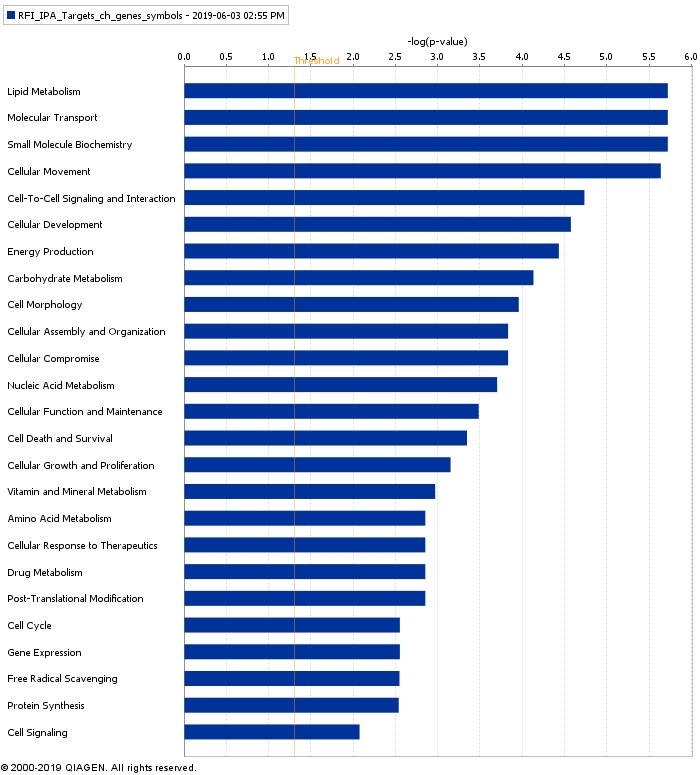


**Figure S11**: All cellular and molecular functions enriched by DE miRNAs’ DE targets (as previously reported by Mukiibi et al. (2018)) at a TargetScan context++ score percentile threshold of greater than 50 in Charolais steers, generated from Ingenuity Pathway Analysis (IPA).


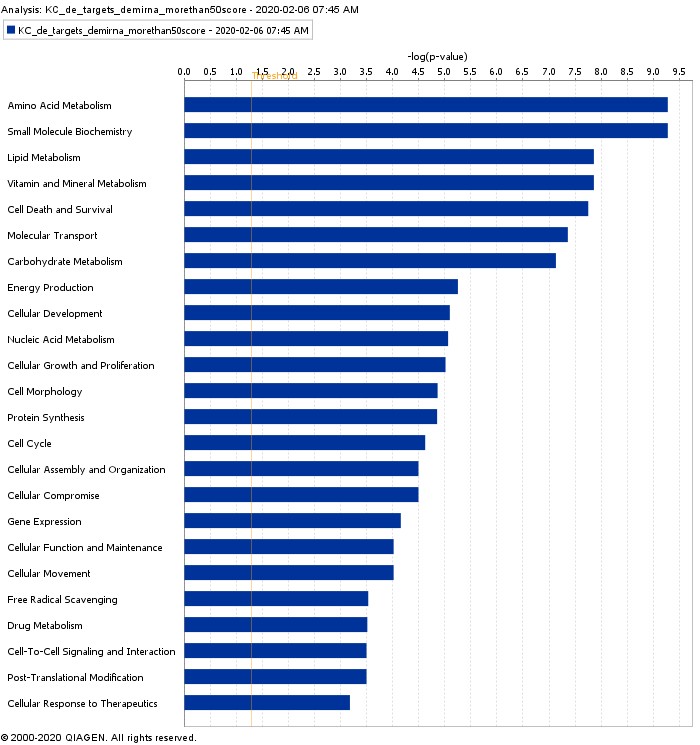


**Figure S12**: All cellular and molecular functions enriched by DE miRNAs’ DE targets (as previously reported by Mukiibi et al. (2018)) at a TargetScan context++ score percentile threshold of greater than 50 in KC steers, generated from Ingenuity Pathway Analysis (IPA).


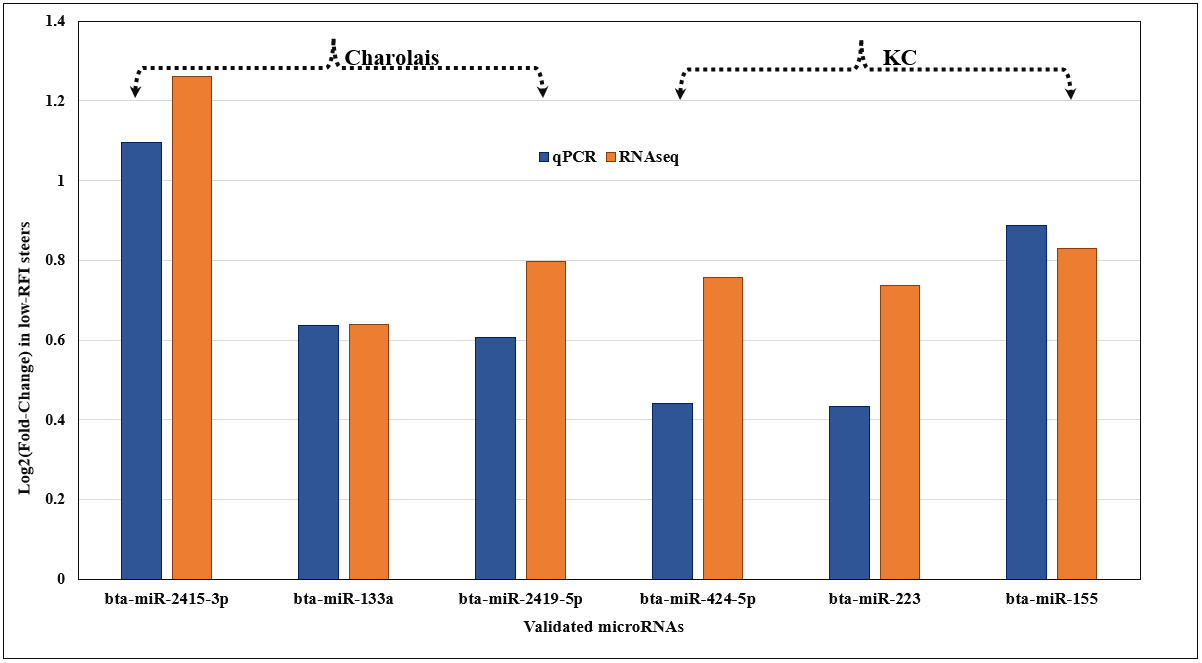


P= 0.086

P= 0.002

P= 0.054

P= 3.55E-05

P= 0.314

P= 4.96E-04

P= 3.62E-04

P= 8.72E-07

P= 0.273

P= 0.003

P= 7.12E-08

P= 0.145

**Figure S13.** Comparison of the expression (in log2(Fold-Change)) of six differentially expressed miRNAs in low-RFI animals as estimated using qPCR (blue bar) and RNAseq (orange bar) methods, P= P-value of expression difference test between high and low-RFI animals. KC: Kinsella Composite.
